# Genetic variations influence brain changes in patients with attention-deficit hyperactivity disorder

**DOI:** 10.1038/s41398-021-01473-w

**Published:** 2021-06-05

**Authors:** Santosh K. Yadav, Ajaz A. Bhat, Sheema Hashem, Sabah Nisar, Madeeha Kamal, Najeeb Syed, Mohamed-Ramzi Temanni, Rakesh K. Gupta, Saddat Kamran, Muhammad Waqar Azeem, Amit K. Srivastava, Puneet Bagga, Sanjeev Chawla, Ravinder Reddy, Michael P. Frenneaux, Khalid Fakhro, Mohammad Haris

**Affiliations:** 1 Laboratory of Molecular and Metabolic Imaging, Sidra Medicine, Doha, Qatar; 2 Department of Pediatrics, Sidra Medicine, Doha, Qatar; 3 Applied Bioinformatics Core, Research Branch, Sidra Medicine, Doha, Qatar; 4 Department of Radiology and Imaging, Fortis Memorial Research Institute, Gurgaon, Haryana India; 5 Neuroscience Institute, Hamad General Hospital, Doha, Qatar; 6 Department of Psychiatry, Sidra Medicine, Doha, Qatar; 7 Department of Pediatric Surgery, McGovern Medical School, University of Texas Health Sciences Center at Houston, Houston, TX 77030 USA; 8 Department of Diagnostic Imaging, St. Jude Children’s Research Hospital, Memphis, TN USA; 9 Department of Radiology, Perelman School of Medicine at the University of Pennsylvania, Philadelphia, PA 19104 USA; 10 Center for Magnetic Resonance and Optical Imaging, Department of Radiology, Perelman School of Medicine at the University of Pennsylvania, Philadelphia, PA 19104 USA; 11 Academic Health System, Hamad Medical Corporation, Doha, Qatar; 12 Department of Human Genetics, Sidra Medicine, Doha, Qatar; 13 Department of Genetic Medicine, Weill Cornell Medical College, Doha, Qatar; 14 Laboratory of Animal Research, Qatar University, Doha, Qatar

**Keywords:** ADHD, Clinical genetics, Molecular neuroscience

## Abstract

Attention-deficit hyperactivity disorder (ADHD) is a neurological and neurodevelopmental childhood-onset disorder characterized by a persistent pattern of inattentiveness, impulsiveness, restlessness, and hyperactivity. These symptoms may continue in 55–66% of cases from childhood into adulthood. Even though the precise etiology of ADHD is not fully understood, it is considered as a multifactorial and heterogeneous disorder with several contributing factors such as heritability, auxiliary to neurodevelopmental issues, severe brain injuries, neuroinflammation, consanguineous marriages, premature birth, and exposure to environmental toxins. Neuroimaging and neurodevelopmental assessments may help to explore the possible role of genetic variations on ADHD neuropsychobiology. Multiple genetic studies have observed a strong genetic association with various aspects of neuropsychobiological functions, including neural abnormalities and delayed neurodevelopment in ADHD. The advancement in neuroimaging and molecular genomics offers the opportunity to analyze the impact of genetic variations alongside its dysregulated pathways on structural and functional derived brain imaging phenotypes in various neurological and psychiatric disorders, including ADHD. Recently, neuroimaging genomic studies observed a significant association of brain imaging phenotypes with genetic susceptibility in ADHD. Integrating the neuroimaging-derived phenotypes with genomics deciphers various neurobiological pathways that can be leveraged for the development of novel clinical biomarkers, new treatment modalities as well as therapeutic interventions for ADHD patients. In this review, we discuss the neurobiology of ADHD with particular emphasis on structural and functional changes in the ADHD brain and their interactions with complex genomic variations utilizing imaging genetics methodologies. We also highlight the genetic variants supposedly allied with the development of ADHD and how these, in turn, may affect the brain circuit function and related behaviors. In addition to reviewing imaging genetic studies, we also examine the need for complementary approaches at various levels of biological complexity and emphasize the importance of combining and integrating results to explore biological pathways involved in ADHD disorder. These approaches include animal models, computational biology, bioinformatics analyses, and multimodal imaging genetics studies.

## Background

Attention-deficit hyperactivity disorder (ADHD) is a clinically heterogeneous neurobiological disorder of inattention, impulsivity, and hyperactivity, affecting 5–7% of children worldwide^1–4^. Severity status and symptoms of ADHD vary throughout a person’s lifespan; however, adult individuals with ADHD show less noticeable signs of hyperactivity and impulsivity than pediatric patients with ADHD^5,6^. ADHD may occur either as an isolated condition or as comorbidity with other neurological, psychiatric, and neurodevelopmental disorders^7,8^. The adverse impact of ADHD on society is profound and multifaceted as it affects not only all aspects of a child’s life but also those of siblings and parents, causing significant disturbances to routine family functioning^9,10^. Furthermore, there are financial burdens related to treatment costs and reduced employment prospects^11^. Based on the severity of the ADHD disorder, it may affect the child’s performance at school, and if not treated or left undiagnosed, it may persist into adulthood, affecting both personal and professional life^11^.

**Fig. 1:**
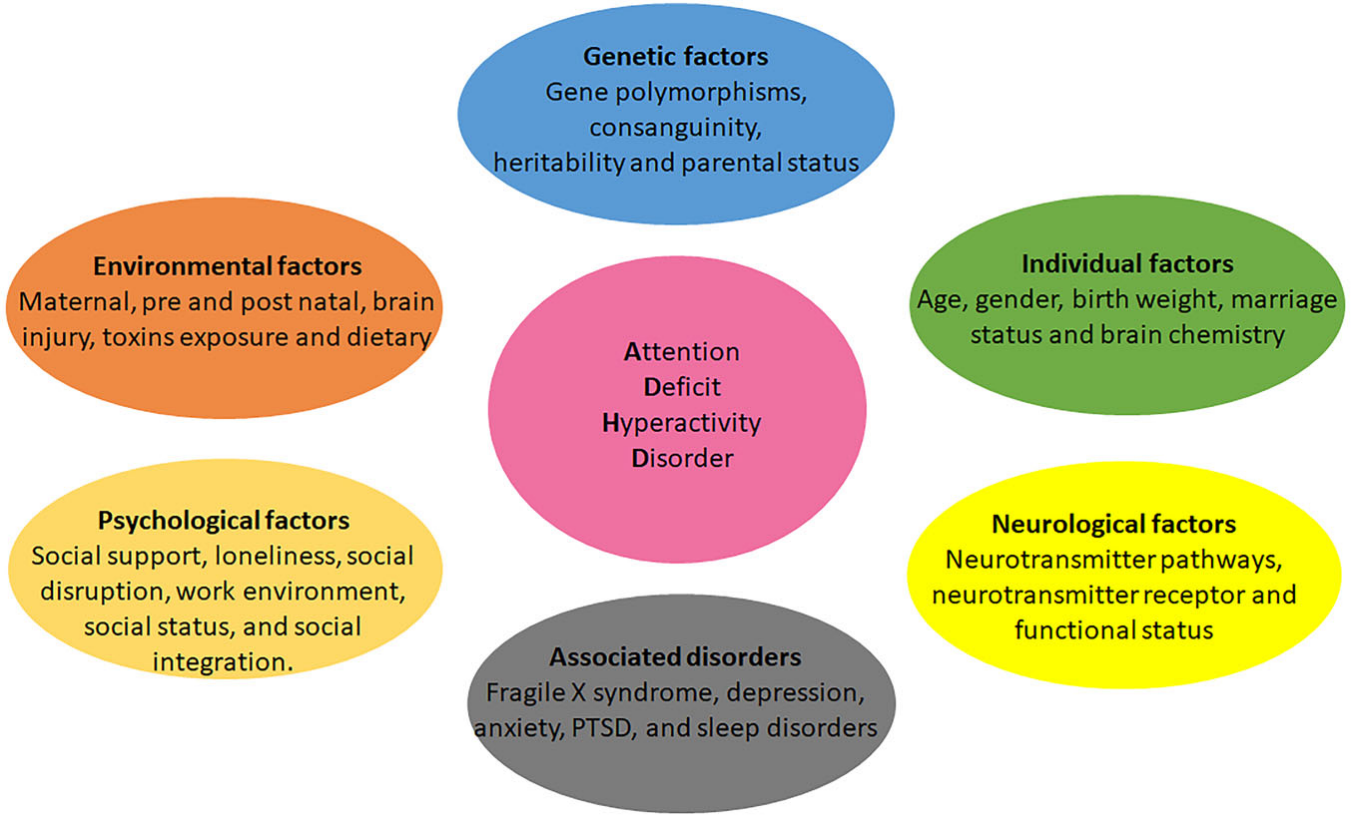
Factors affecting ADHD pathophysiology. Different factors such as genetic, in particular, gene polymorphisms, environmental factors, psychological factors, individual factors such as age, abnormality in various neurological pathways such as dopaminergic and serotonergic, and comorbidity with multiple disorders are associated with symptoms of attention-deficit hyperactivity disorder.

Multiple factors contribute to ADHD symptoms, including genetic predisposition, neurodevelopmental issues, abnormal neuronal maturation, brain injury, environmental exposures, and consanguineous marriages. A recent study by Posner et al. reported that environmental risk factors during prenatal, perinatal, and postnatal stages contribute to the symptom of ADHD. The prenatal and perinatal risk factors, including premature birth, low birth weight, history of maternal exposure to tobacco, stress, trauma, and obesity, are substantially associated with ADHD. Postnatal risk factors such as trauma, parenting style, artificial coloring, and flavoring food agents, exposure to pollutants and pesticides can exacerbate the symptoms of ADHD^12^ (Fig. 1).

Despite advancements in the diagnosis of various neurological and psychiatric disorders, the accurate and early diagnosis of ADHD still poses a considerable challenge. According to the American Psychiatric Association Diagnostic and Statistical Manual, Fifth Revision (DSM-V), the current diagnostic criteria for ADHD include subjective measurements of inattentiveness (six or more symptoms of inattention) and hyperactivity and impulsivity (six or more symptoms of hyperactivity–impulsivity)^4^. This subjective assessment in the decision-making process may promptly overestimate or underestimate the symptoms of ADHD, especially in the pediatric population resulting in both overdiagnosis and misdiagnosis.

Clinical symptoms of ADHD are associated with aberrant structural and functional changes in the brain^13^. Several neuroimaging studies have been performed to evaluate the anatomical, microstructural, functional, biochemical, and molecular changes in the brains of ADHD^14–16^. Numerous neural networks implicating attention, executive and reward functions, and molecular pathways such as dopaminergic, adrenergic, serotonergic, and cholinergic have been identified that play a critical role in the pathophysiology of ADHD, and these pathways can be studied in great details because of the advances in the field of neuroimaging^17,18^. Molecular genomic studies, on the other hand, have observed a strong genetic influence on ADHD with a heritability estimate rate between 70 and 90%^19–21^. Several candidate genes, variants, and chromosomes associated with ADHD symptoms have been found in multiple studies focussing on correlation, linkage, and meta-analysis investigating the genetic susceptibility of ADHD. Genome-wide association studies (GWAS) have found genomic DNA copy number variants and rare or large deletion/duplications in ADHD^22,23^. Studies using fine-mapping linkage analysis found variation in the gene encoding for neuronal signaling as a potential risk factor for ADHD^24–26^. Multiple gene polymorphisms showed an association with changes in the neuropsychobiological functions in ADHD^27,28^. Most of the individual candidate gene association studies in ADHD have been conducted on a small sample size and the reported variants were not strongly associated with the ADHD pathology. Also, a recent genome-wide association (GWAS) based meta-analysis study, performed in a significantly large sample size, did not observe any association between previously reported candidate genes and ADHD^29^.

Structural and functional imaging-derived phenotypes can be used to understand neuropsychobiology of the disorder caused by genetic changes, and this can lead to a better understanding of the clinical presentation of ADHD phenotypes. Intermediate phenotypes are those characteristics of a disorder that are correlated explicitly with its neurobiology than clinical features and contribute to the disorder’s genetic susceptibility itself. Phenotypes derived from neuroimaging, such as structural, functional, and molecular characteristics considered to be of high inheritance, are shown to be significantly altered in ADHD and found to be vital intermediate phenotypes for ADHD. This review aims to provide an overview of the structural and functional changes in the ADHD brain and their interactions with complex genomic variations. We also discuss the requirement for combinatorial methodologies that can be achieved by combining neuroimaging-derived phenotypes with genomic components to better understand ADHD neuropsychobiology.

## Brain changes in ADHD

The behavioral/cognitive profiles in ADHD are likely to be controlled in early childhood by the structural, functional, and molecular brain changes. Many characteristic attributes, such as gene mutations/polymorphisms, neural development, neuronal maturation, neuronal functions, cortical and subcortical structures, metabolite levels, brain blood flow, and connectivity patterns, are abnormal in the brain of ADHD patients^30–37^. Changes in the ADHD brain can be classified into various categories, as described in more detail in the following sections.

### Structural brain changes in ADHD

#### Gray matter and subcortical changes

Magnetic resonance imaging (MRI) is a widely used noninvasive modality for mapping in vivo brain changes in various neurological disorders. Whole-brain volumes, mainly gray and white matter volumes, have been used to differentiate patients with ADHD from healthy controls. T1-weighted high-resolution brain imaging provides superior contrast between the brain tissues and is frequently used to measure brain tissue changes, including gray matter and subcortical regions in different neurological disorders, including ADHD. MRI studies in patients with ADHD have shown lower overall brain gray matter volume (3–5%) than controls^31,38–40^. Several meta-analyses studies using automated voxel-based morphometric analysis have been performed to explore the global and regional gray matter volume changes in ADHD^41–44^. A meta-analysis, based on 14 studies, observed that patients with ADHD demonstrated a reduced overall volume of gray matter, particularly in the right caudate and lentiform nuclei^43^.

Furthermore, a meta-analysis involving 931 patients with ADHD and 822 control subjects found a reduction in the volume of gray matter in bilateral basal ganglia and insular regions in ADHD patients compared to controls^44^. A cross-sectional MRI study showed a reduction in global brain volume by 2.5% and total gray matter volume by 3% in ADHD patients than control. These patients have shown significantly lower caudate nuclei and putamen volumes on regional brain analysis^31^. Another study relatively conducted on a large number of subjects including 307 subjects with ADHD, 169 siblings without ADHD, and 196 healthy controls reported lower gray matter volume in 5 brain sites, including the medial and orbitofrontal cortex, precentral gyrus, and para cingulate cortices in the ADHD group. In comparison, siblings without ADHD exhibited a pattern of lower volumes of gray matter in all brain sites except the precentral gyrus compared to healthy controls^45^.

Multiple studies were conducted to identify neurodevelopmental changes by analyzing cortical thickness, surface area, subcortical volume, and gyrification index in ADHD^31,37,40,42,43,45–47^. A higher degree of gyrification is necessary to maximize the cortical surface area while preserving the compact brain size. The gyral and sulcal folding in the brain relates to the efficient corticocortical connections and neuronal fibers compaction. The abnormal cortical folding signifies deficits in structural and functional connectivity. Several studies have reported changes in cortical thickness, showing both increased and decreased cortical thickness in ADHD^32,48–55^. A longitudinal study found a lower global cortical volume in ADHD patients, driven mainly by a reduction in the volume of the front lobe associated with a smaller surface area and gyrification, and almost all changes remained significant throughout the development^56^. Another study showed a synchronized delay in cortical thickness growth^32^ and surface area in children with ADHD, suggesting that there could be an overall slow cortical maturation in ADHD that could lead to abnormal neural functions^33^. Normal brain development in early childhood tends to increase grey matter volume, and throughout neuronal development, populations are pruned to provide optimal functional efficiency. Alterations in gray matter or cortical thickness reflect abnormal neuronal maturation. Shaw et al. observed a delay of 2–3 years in cortical thickness development in both motor and sensory cortices in children with ADHD compared to age-matched controls^33,38^. The surface area, another important parameter to assess brain maturation, has been found to be associated with developmental delay in ADHD, predominantly in the right prefrontal cortex^33^. A cross-sectional meta-analysis study on children and adults with ADHD (age range: 4–63 years) using enhancing neuroimaging genetics through meta-analysis observed significant smaller subcortical volumes from multiple brain regions such as caudate, putamen, accumbens, amygdala, hippocampus, and reduced total intracranial volume in ADHD relative to healthy controls^37^. Collectively, these studies suggest that the brain is altered in a more widespread manner in ADHD than has been previously hypothesized.

#### White matter changes in ADHD

White matter tissue makes deep brain regions and consists of bundles of myelinated axons. It is organized into tracts and primarily involved in coordinating communication between different brain areas. Intact white matter is responsible for improved learning and brain functions. Various neurological and psychiatric disorders, including ADHD, are associated with white matter abnormalities, as revealed by several diffusion tensor imaging (DTI) studies^34,35,57–59^. DTI is an MRI-based technique used to characterize the microstructural tissue integrity (nature and extent of neuronal disruption) and microfiber pathways by using diffusion properties of water molecules. Fractional anisotropy (FA) measures the directionality of water diffusion of the underlying tissue structures, while mean diffusivity (MD) estimates the magnitude of water diffusion in tissue. Both FA and MD are the most frequently used DTI metrics for quantitative estimation of the white matter integrity in various neurological and neurodegenerative disorders. Greater FA values are associated with higher directionality of diffusion, especially in white matter regions, and may suggest intact axonal integrity, while higher MD values are related to loss of myelin and tissue’s integrity. Other DTI-derived metrics such as axial diffusivity and radial diffusivity provide valuable information about the degree of axonal integrity and myelination^60–62^. A meta-analysis based on DTI findings observed significant microstructural tissue abnormality mainly in the white matter areas of the frontostriatal-cerebellar neurocircuitry in ADHD compared to healthy participants^34^. It has been reported that decreased FA in the corpus callosum of adult patients with ADHD was attributed to deviations in radial diffusion instead of axial diffusion, indicating abnormal myelination process may be a dominant factor for poor neurobiological performance in adult patients with ADHD^35^. A study on an adolescent patient with ADHD showed lower FA and higher radial diffusivity in multiple brain sites, including corpus callosum and major fiber tracts in the left hemisphere, and the lower FA values were correlated with the inhibition performance in ADHD^57^. Another tract-based analysis performed in relatively bigger sample size on adult patients with ADHD showed reduced FA and increased MD and radial diffusivity in various brain regions^35^. A meta-analysis of DTI-derived tract-based spatial statistics observed reductions in FA from corpus callosum regions that extended to the right cingulum region, the left tapetum, and the right sagittal stratum. This study also reported that the reduced FA in splenium was negatively correlated with the age of ADHD patients^58^. Using whole-brain voxel-based morphometry analysis, a recent meta-analysis documented both decreased and increased FA from multiple brain regions in patients with ADHD compared to typically developing (TD) children^59^. Integrating the structural imaging-based morphometric and diffusion tensor imaging-based tractography, it has shown that the altered fronto-accumbal circuit was associated with a higher frequency of aggression in ADHD children. These findings implicate an important role of the fronto-accumbal circuit in the pathophysiology of ADHD, which can be further explored to treat aggressive behaviors in ADHD children^63^.

### Functional brain changes in ADHD

Functional MRI (fMRI) is a neuroimaging technique widely used to measure brain activity in vivo^64–68^. The fMRI quantifies the cerebral activity based on oxygen consumption by active neuronal cells, resulting in a shift in the blood oxygenation level. The fMRI can be used to measure changes in the neuronal activity against a specific task and at the resting-state fMRI (rs-fMRI). fMRI studies have reported altered neuronal signals in multiple brain sites, especially in the prefrontal and cerebellum region in patients with ADHD^69–76^. Task-based fMRI is performed mainly to explore brain activity against a specific task. In response to working memory, inhibitory control, and attentional tasks, ADHD patients showed lower activation in frontostriatal, parietal, and attentional networks than healthy controls^69^. A meta-analysis fMRI study against attention and inhibition tasks showed functional abnormalities in two different domains connected to fronto-basal-ganglion networks in ADHD patients^70^. The fMRI study based on reward task demonstrated decreased activation in the striatum brain region in ADHD patients compared to control^71^. Another reward task-based fMRI study observed increased activation in the anterior cingulate, anterior frontal cortex, cerebellum, orbitofrontal, occipital cortex, and ventral striatum in children with ADHD compared to control^72^. The meta-analysis study related to time-based task showed decreased activation in the brain area responsible for the timing, including the insular, cerebellum, and the left parietal lobe, in patients with ADHD compared to healthy controls^70^. Recently, a stop-signal task-based fMRI study showed a significant hypoactivation in the left superior frontal, inferior frontal, medial frontal, and bilateral temporal and parietal areas in children with ADHD compared to siblings without ADHD and healthy participants, suggesting hereditary patterns in the activation.

The rs-fMRI is considered a powerful technique and has recently had considerable interest in ADHD neuroimaging studies. It has been shown that at resting-state spontaneous fluctuations in blood oxygenation level were observed in the human brain without any functional task^77^. Multiple metrics are quantified for rs-fMRI, including regional homogeneity (ReHo) and amplitude of low-frequency fluctuation (ALFF). The ReHo quantifies the regional similarity of the brain activity, while ALFF measures the brain signal variability of a given voxel. The rs-fMRI showed brain abnormalities in various domains, including sensorimotor, default mode network, cerebellum, cortex, anterior cingulated cortex, and other related brain sites in ADHD patients^78^. Abnormal global and local brain neural activity has emerged as discerning parameters to distinguish ADHD from healthy subjects^79,80^. It has been reported that atypical resting-state functional connectivity belongs to the cortical–striatal–thalamic circuitry^73,74^, and this connectivity is mostly associated with the neuropsychological status of ADHD^75,76^. Another study demonstrated that changes in the default mode network correlated with the behavioral changes in ADHD^81^. Emotion regulation is a common issue in children with ADHD and is shown to be associated with altered amygdala–cortical resting-state functional connectivity^82^.

In a recent study, Tan et al. combined pseudocontinuous arterial spin labeling and rs-fMRI techniques simultaneously to study alterations in cerebral perfusion and functional connectivity in a cohort of medication naïve male adults with ADHD. The study observed several interesting findings that may help to comprehensively understand the neuropathogenesis of ADHD. First, a significant reduction in cerebral blood flow (CBF) from subcortical regions and other regions involved in functional networks such as somatomotor network, ventral attention network, the limbic network was observed compared to those of age-matched healthy controls^83^. It was further suggested that the possible cause of hypoperfusion and abnormal vascular response in patients with ADHD could be attributed to impaired dopamine and nitric oxide systems^84^. Second, a lateralization trend was observed, as hypoperfusion areas were located primarily in the left cerebral hemisphere. Some earlier studies^85,86^ have also reported atypical lateralization (abnormal right > left asymmetries) in ADHD patients with altered CBF and brain connectivity observed from the left hemisphere. Taken together, these findings indicate that one potential component of ADHD is the over-aroused right cerebral hemisphère.

Another notable finding was that significant negative associations with severity of the disorder were found between the CBF in the left amygdala, hippocampus and the left putamen, global pallidum. In addition, the left amygdala showed significantly decreased functional connectivity with the prefrontal cortex (PFC). As the amygdala is involved in “bottom‐up” (support or influence emotion regulation) emotional processing and PFC is vital for the emotional processing in “top‐down” (attention to emotionally arousing stimuli) regulation^87^, the disrupted connectivity between these two regions support a notion of emotional dysregulation that is generally observed in patients with ADHD. Collectively, these findings provide valuable insights into the pathophysiological mechanisms of neurovascular coupling in ADHD.

### Magnetic resonance spectroscopy (MRS) detectable brain metabolites alterations in ADHD

Magnetic resonance spectroscopy (MRS), a noninvasive imaging technique, provides a quantitative assessment of in vivo brain metabolites. Proton MRS (^1^H MRS) is the most commonly used MRS technique to quantify the different metabolites such as *N*-acetyl aspartate (NAA), choline (Cho), glutamate/glutamine (Glx), creatine (Cr), and myoinositol (mI) by targeting their aliphatic protons (−CH2 or −CH3). Collectively, these metabolites play an essential role in maintaining the brain’s structures and regulating various physiological processes; for example, NAA is considered as a marker of neuronal integrity and viability, and reduced NAA levels indicate neuronal loss. Cho is a cell membrane marker, and its elevation indicates increased turnover of cell membranes and the lower choline levels are associated with demyelinating processes. Glutamate/glutamine (Glx) are neurotransmitters of the glutamatergic system and play a key role in neuronal signaling, neuronal maturation, proliferation, and migration. Changes in the levels of Glx are associated with abnormal neurophysiology of the brain. Cr is mainly involved in phosphate metabolism and responsible for energy consumption and storage. Several ^1^H MRS studies have reported significant alterations in brain metabolism in ADHD patients. For instance, children with ADHD had higher levels of Glx from the frontalstriatal and right dorsolateral frontal region than healthy controls^88^. Also, adults with the combined type (both inattention and hyperactivity/impulsivity) of ADHD were found to have significantly reduced NAA levels in the dorsolateral prefrontal cortex relative to the inattentive type of ADHD patients and control subjects^89^. In addition, higher levels of Glx in the anterior cingulate cortex were observed in children with ADHD compared to children with bipolar disorder and healthy controls^90^. However, ^1^H MRS study from a large population of adult patients with ADHD found significantly lower Glx levels from the right anterior cingulate than healthy controls^91^. The difference in the Glx level between pediatric and adult patients with ADHD indicates that age is a key factor that regulates excitatory/inhibitory neurobiological activities in ADHD. A meta-analysis study reported increased levels of Cho from the prefrontal cortex, striatum, and anterior cingulate cortex in ADHD patients compared to healthy controls^92^.

A recently developed technique known as GluCEST^93^ can be utilized to generate high-resolution parametric maps of glutamate to study its role in various neurological disorders, including ADHD^94–97^. GluCEST detected decreased glutamate levels in the brain of the Alzheimer’s mouse model^97^, while higher GluCEST contrast in the brain of the Parkinson’s mouse model was observed^96^. Similarly, GluCEST mapped the changes in the glutamate level in the brain of patients with schizophrenia and temporal lobe epilepsy^98,99^. So far, there is no study of GluCEST imaging in patients with ADHD, we suggest that the GluCEST technique can be beneficial to evaluate the effect of the gene on the brain’s glutamate level in ADHD patients.

## Genetic changes in ADHD

**Fig. 2:**
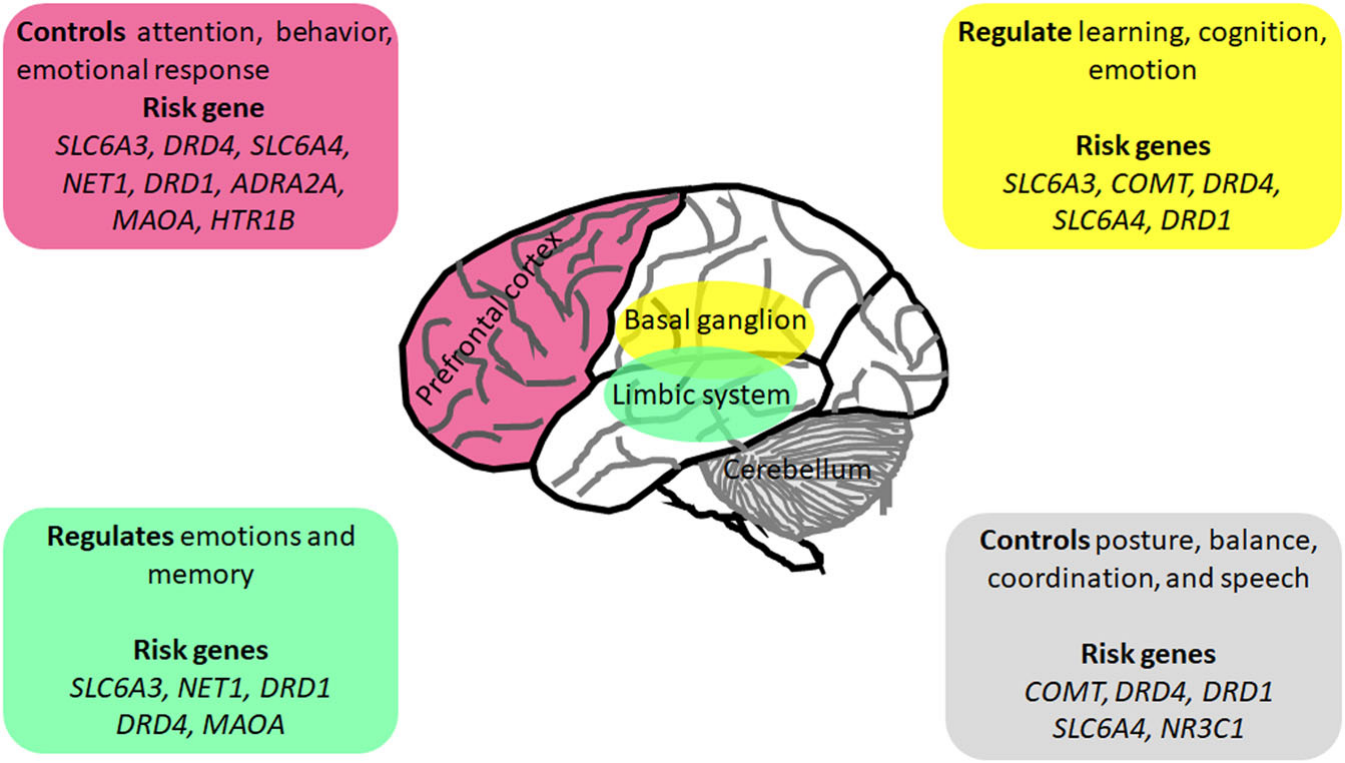
Risk genes and associated altered brain regions in attention-deficit hyperactivity disorder. Multiples genes are associated with altered structural and functional brain changes, mainly in the frontal lobe, basal ganglion, limbic system, and cerebellum.

Studies on the family, sibling, and adoption indicate that ADHD has significant genetic components. First and second-degree ADHD families are at higher risk for the disorder^100^. Different molecular genetic studies have been conducted to identify ADHD risk genes (Fig. 2).

**Fig. 3:**
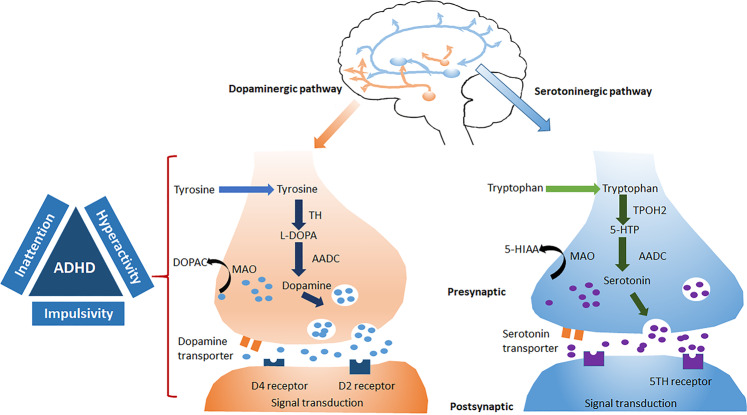
Major pathways related to the pathogenesis of attention-deficit hyperactivity disorder (dopaminergic and serotonergic). Dopaminergic and serotonergic neurons are primarily located respectively in the midbrain and hindbrain and control various functions. Anomalies in dopamine and/or serotonin levels contribute to the symptoms of inattention, hyperactivity, and impulsiveness in attention-deficit hyperactivity disorder (Figure inspired from the manuscript by Fontana BD et al., 2019^187^).

Numerous meta-analysis studies have found significant relationships in the multiple genes for common genetic polymorphisms/variants^101–103^. Though several multiple twin studies found that heritability estimates in ADHD could reach up to 90%^19,20,104^, it is still challenging to identify ADHD risk genes,^101,105^ due to the complex and polygenic nature of ADHD pathophysiology. Besides genetic factors, many external risk factors, such as environment and possible interactions between gene and environment, are also associated with the increased risk of ADHD^104^. Genes encoding for dopamine and serotonin transporters are associated with ADHD (Fig. 3)^101–103^.

An International Multi-site ADHD Genetics (IMAGE) project performed an in-depth analysis of more than 50 candidate genes from European multicenter samples of around 674 families showed significant associations of several candidate genes with ADHD^106^. Another meta-analysis based on seven linkage studies showed that the small arm of chromosome 16 might be associated with ADHD symptoms^107^. A study using cadherin 13 (*CDH13*) knock-out mouse model observed that *CDH13* modulates the synaptic activity of hippocampal interneurons and cognitive domains and it was suggested to be a risk gene for ADHD^108^. A linkage analysis-based study on multigenerational families identified the adhesion G protein-coupled receptor L3 (*ADGRL3*) gene (previously known as *LPHN3*) variants susceptibility in developing ADHD^109^. Another GWAS found enrichment of rare copy number variants^110^, such as *CHRNA7, NPY* genes, and genes encoding for glutamate receptors, suggesting that rare variant involvement in ADHD neuropsychobiology is very mixed and is similar to a common variant contribution^22,111^. ADHD-related genes are spread across the entire genome but, as found on clustering analysis, they tend to be clustered into specific functional groups. A study using group comparison enrichment analyses observed that enriched functions for the ADHD-GWAS association were linked to neuronal projections, synaptic structures, nervous system structures, neuronal morphogenesis, cell–cell interaction, glutamatergic signaling^112,113^. Another GWAS on five common psychiatric disorders, including ADHD, from Psychiatric Genomics Consortium, found the association of calcium channel signaling genes with multiple psychiatric and neurological disorders, suggesting that gene variants in calcium channel activity may have pleiotropic effects in the evolution of ADHD neuropsychobiology.

Due to their strong effect on ADHD genetics, the risk genes identified in ADHD are associated with multiple processes, and some of them are known as hot genes. Hot genes are defined as candidate genes reported by at least five studies. Currently, 24 hot genes represent the top 7% of ADHD candidate genes^114^. Such genes are involved in many neurobiological processes, including neurotransmitter biosynthesis, modulation of synaptic membrane dynamics, glutaminergic signaling, and various transcriptional mechanisms.

**Fig. 4:**
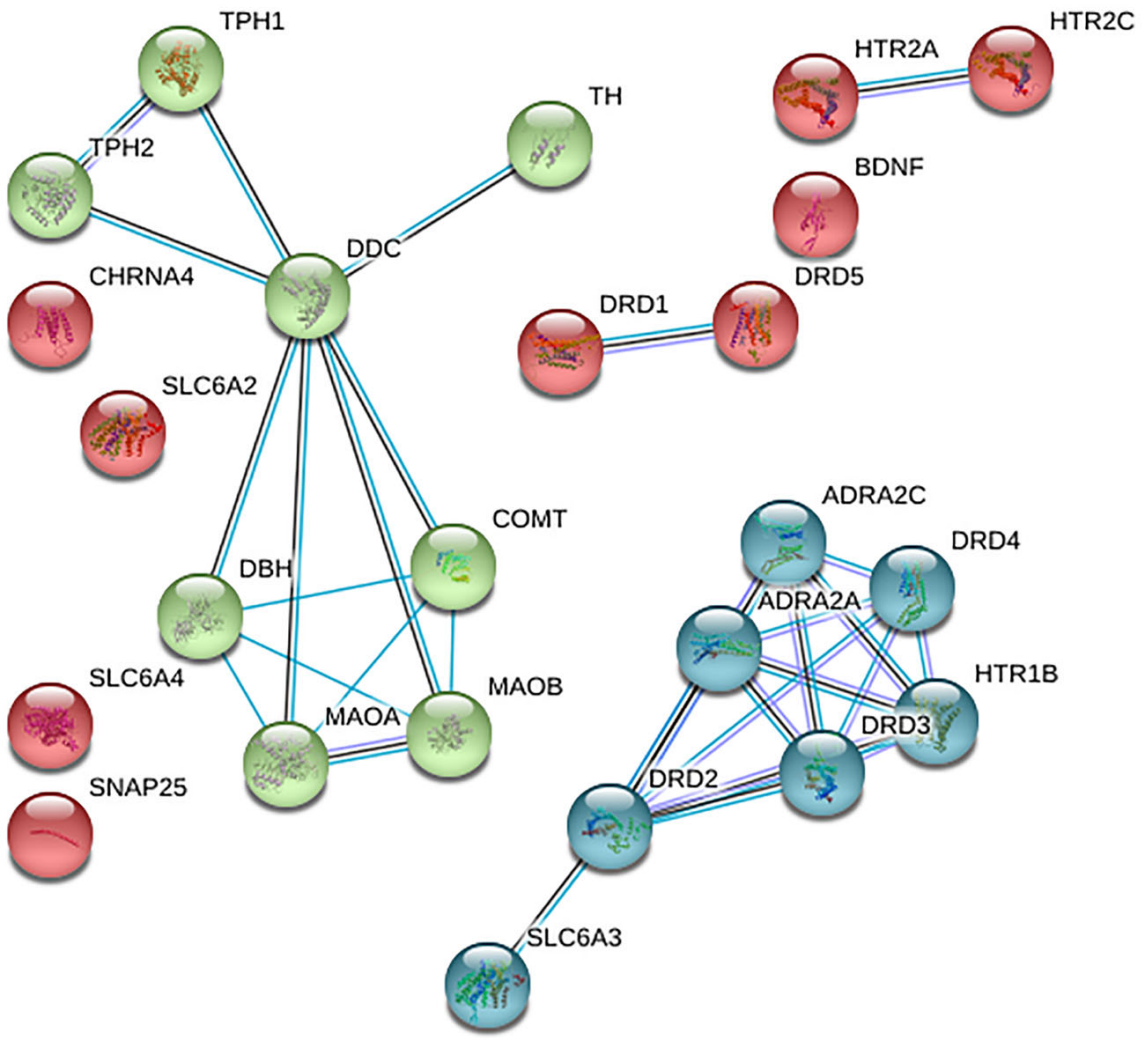
Interaction map of hot genes associated with various parts of brain changes as observed on structural and functional MRI in attention-deficit hyperactivity disorder, generated using STRING1 webserver. All sources are used to create the interaction model with a default medium confidence interaction score of 0.4. and *k*-means clustering method. The line color indicates the type of interaction evidence: blue line denotes co-occurrence, black line indicates co-expression, and the purple line indicates experimental evidence ref: https://string-db.org/.

We analyzed these genes in known interacting biological networks and for enrichment in biological pathways. We also examined the expression patterns of candidate genes across brain regions and synaptic, presynaptic membranes. First, we performed a gene network interaction analysis of 24 hot genes using STRING1 (search tool for recurring instances of neighboring genes) webserver. By an iterative approach, this server retrieves the genes that are indirectly (via other genes) associated with the query gene. The web interface (https://string-db.org) visualizes the results in their genomic context (Fig. 4). Our analysis identified a total of three gene clusters (red, green, and blue) based on *k*-means clustering with an edge confidence value of 0.015 indicates no connection and a value of 0.90 indicates highly connected genes (Fig. 4). Thirteen genes dopamine receptor D3 (*DRD3*), adrenoceptor alpha 2A (*ADRA2A*), dopa decarboxylase (*DDC*), dopamine receptor D2 (*DRD2*), 5-hydroxytryptamine receptor 1B (*HTR1B*), adrenoceptor alpha 2C (*ADRA2C*), dopamine receptor D4 (*DRD4*), monoamine oxidase A (*MAOA*), monoamine oxidase B (*MAOB*), catechol-*O*-methyltransferase (*COMT*), dopamine beta-hydroxylase (*DBH*), tryptophan hydroxylase 1 (*TPH1*)*,* and tryptophan hydroxylase 2 (*TPH2*) out of 24 hot genes showing five or more than five connections, considered as hub genes. These clusters of genes are more connected to one another than they are to other groups of genes and thus can help identify functional modules. These genes are involved in various biological processes, molecular functions, cellular components displaying polymorphisms, and maybe the potential risk factors for ADHD (Fig. 4).

**Fig. 5:**
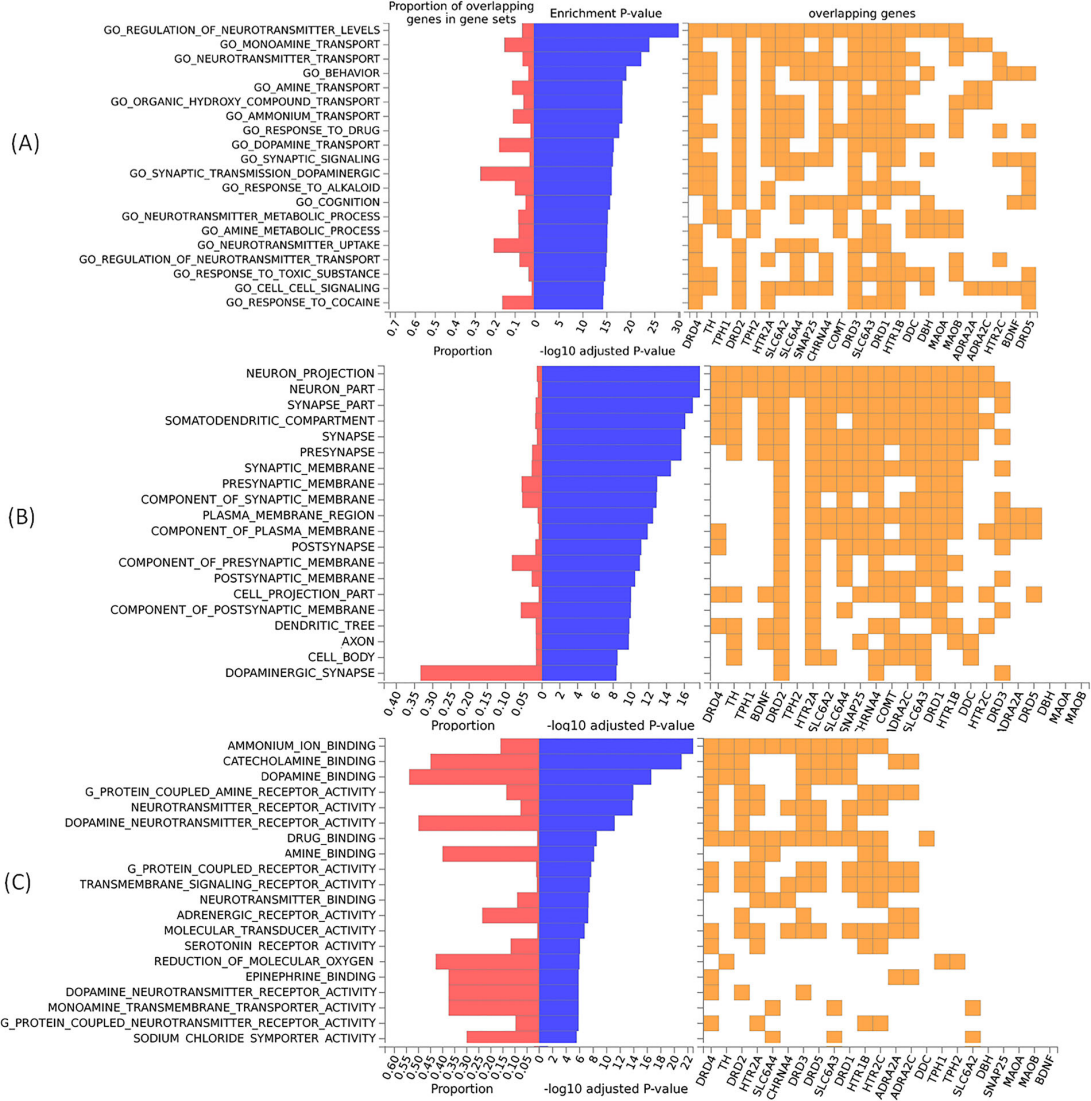
Enrichment analysis of hot genes which predispose to ADHD. Summary of the top 20 gene ontology (GO) in terms of biological processes (**A**), cellular components (**B**), and molecular functions (**C**). The proportion represents the number of genes enriched in each GO category. Significant enrichment genes belong to neurotransmitters (**A**), neuron projections, part of neurons (**B**), and ammonium ion (**C**), which play a vital role in synaptic interactions suggesting the risk factor for ADHD is polymorphism in the enriched genes.

Next, we used all 24 hot genes to perform an enrichment analysis workflow using molecular signatures database (MSigdb) datasets grouped according to gene ontology (GO) categories related to biological processes, cellular components, and molecular functions. The MSigDB is one of the most widely used and comprehensive databases of gene sets for performing gene set enrichment analysis. For the study, a *P* value of 0.05 was used as a cutoff with a minimum of the two overlapping genes selected with gene sets. To functionally annotate genes that are enriched in different biological processes, cellular components, molecular functions, and enrichment of differentially expressed genes, GENE2FUNC, a core process of FUMA (Functional Mapping and Annotation of Genome-Wide Association Studies) (http://fuma.ctglab.nl/), was employed. In the case of ADHD, a set of 24 genes was used as input. Gene enrichment analysis revealed that in the biological processes, the significant enrichment with maximum threshold was observed in neurotransmitter signaling (*P* = 10^−28^) with 19 overlapping genes (19/24); in cellular components, the significant enrichment with maximum threshold was observed in neuron projections (*P* = 10^−16^) and neuron part (*P* = 10^−16^) with 18 overlapping genes (18/24) in both the components. In contrast, in molecular functions, the significant enrichment with maximum threshold was observed in ammonium ion binding (*P* = 10^−22^) with 12 overlapping genes (12/24) (Fig. 5).

The presence of highly significant enrichment genes belongs to neurotransmitters, neuron projections, part of neurons, and ammonium ion binding, which all play a vital role in synaptic transmission, suggesting that the risk factor for ADHD is polymorphism in enriched genes.

**Fig. 6:**
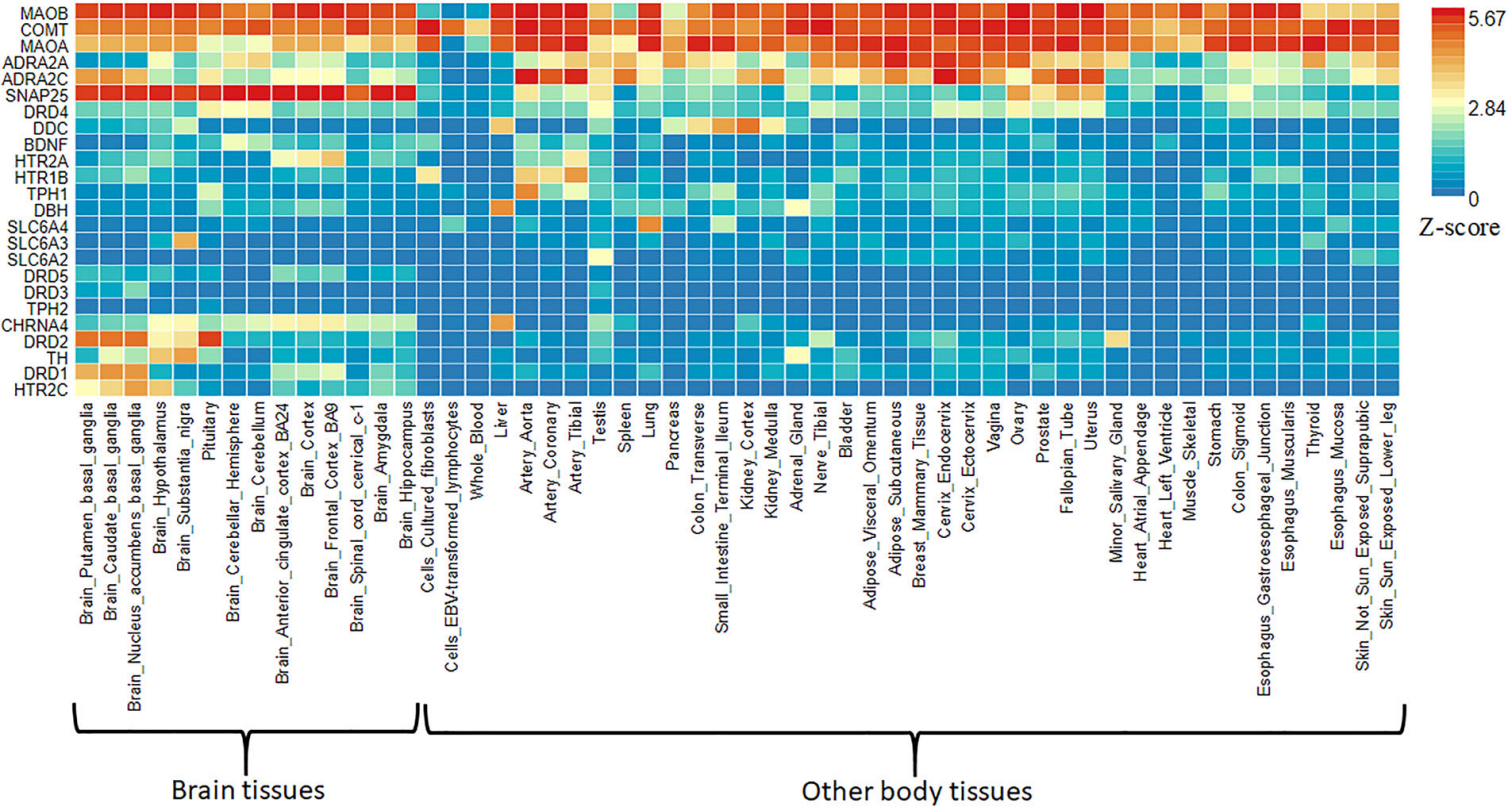
Gene enrichment analysis of hot genes associated with ADHD. Gene expression heatmaps constructed with GTEX v8 (54 tissue types). The heatmap indicates the significance of expressed gene modules related to brain regions. Blue to red reflects a significant association of the gene with brian regions as determined by a standardized *z* score. The gene expression heatmap showing higher relative expression levels of *MAOB, SNAP25, COMT, MAOA, ADRA2C, DRD1, DRD2, HTR2C, CHRNA4*, and *TH* in different brain sites suggest that these genes may be linked with brain areas and are considered as a risk factor for ADHD.

**Fig. 7:**
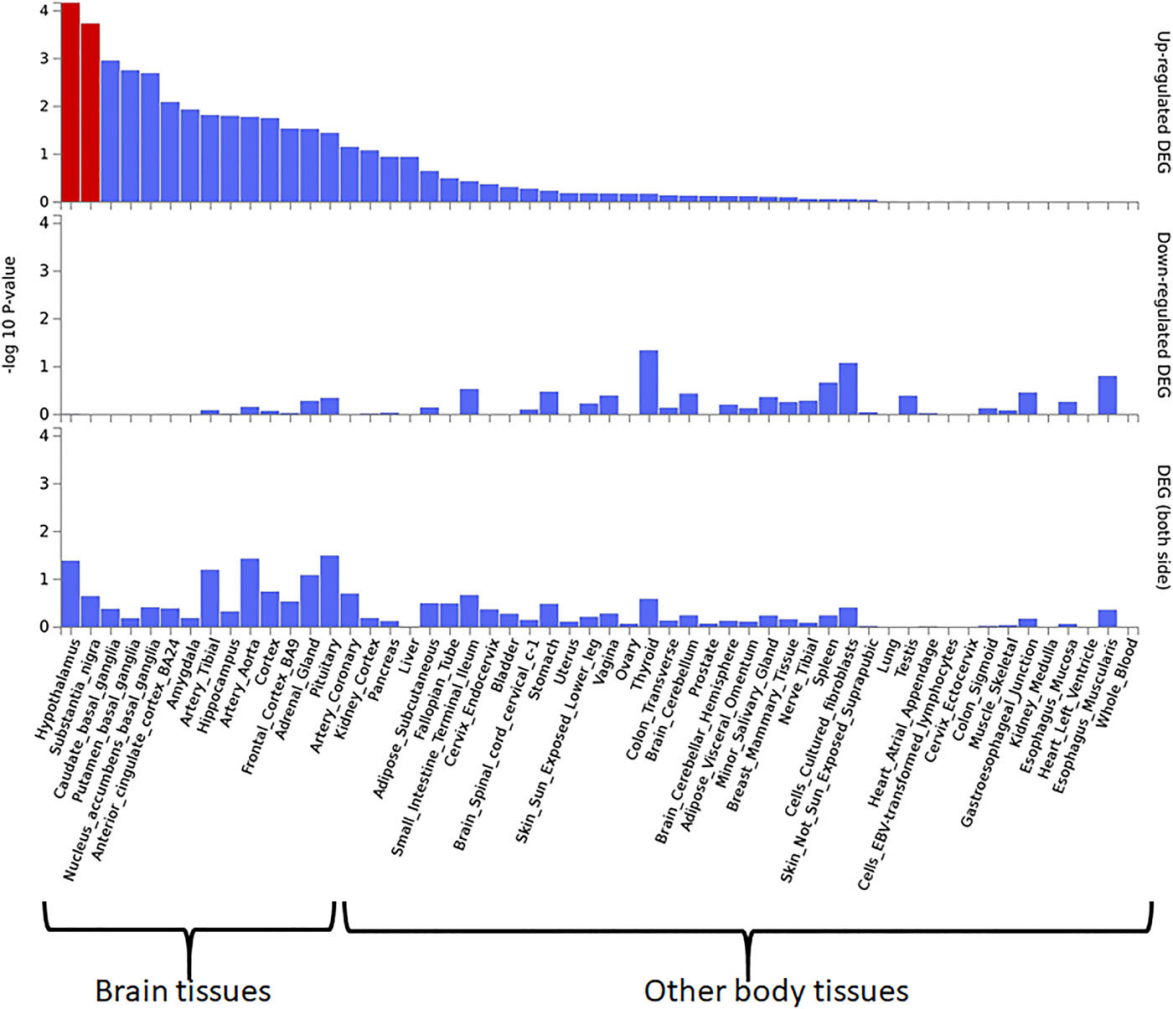
Differently expressed gene (DEG) plots of 24 ADHD hot genes constructed with GTEX v8 across 54 tissue samples. Significantly enriched differently expressed gene sets, highlighted in red, belong to the hypothalamus and substantia nigra.

ADHD gene expression heatmap was created with GTEX v8 (54 tissue types). Heatmap shows standardized expression value (zero mean normalization of transformed expression log2) with dark-red displaying maximum relative expression of that gene in each band, compared to a dark blue color (Fig. 6). A two-sided Student’s *t* test was performed against the remaining bands (types of tissue) per gene per tissue, and a *P* value < 0.05 was considered significant after Bonferroni correction. ADHD gene expression heatmap revealed higher relative expression levels across several brain tissues for the following genes; *ADRA2C, COMT*, dopamine receptor D1 (*DRD1*), *DRD2, MAOA, MAOB*, synaptosome associated protein 25 (*SNAP25*), and tyrosine hydroxylase (*TH*), suggesting that these genes may be considered as a risk factor for ADHD. We also performed the differently expressed gene (DEG) analysis for ADHD hot genes using GTEX v8 (54 tissue types). The DEG analyses showed significant upregulation of ADHD hot genes in the brain sites, including hypothalamus and substantia nigra suggesting the tissue-specific gene expression pattern could be associated with ADHD (Fig. 7).

A recent GWAS study by Demontis et al. in a large sample size observed 12 independent loci surpassing the genome-wide significance threshold. The study identified three candidate genes (FOXP2, SORCS3, and DUSP6) in the association regions^29^. The *FOXP2* gene is located on chromosome 7, which encodes a forkhead/winged-helix transcription factor, characterized by a 100-amino acid monomeric DNA-binding domain and plays a critical role in the synapsis formation and neuronal mechanisms related to speech and learning^115,116^. The *SORCS3* gene, located on chromosome 10, encodes for transmembrane receptors and is high expresses in the brain. This gene is important for neuronal development and plasticity^117^. On the other hand, the *DUSP6* gene is located on chromosome 12 and encodes for the dual-specificity phosphatase 6 enzyme and regulates neurotransmitter homeostasis by affecting the dopamine level in the synapses. Though *DUSP6* expresses at a low level in the brain but plays a critical role in brain development^118–120^.

## Association of brain changes with genetics in ADHD

Advancement in imaging and molecular genomic technologies offers the opportunity to examine the impact of the genetic variations on the structural, functional, and neuronal connectivity of the brain along with the study of dysregulated biological pathways in various neurological and psychiatric disorders. Using neuroimaging genomics studies, a large body of work has demonstrated a significant association of structural and functional brain changes with genetic variations in ADHD (Tables 1 and 2). Recent developments in imaging and genomics allow a more in-depth investigation of the neurobiological pathways involved in ADHD. Such research is appropriate to understand the relationship between neurodevelopmental and neurofunctional changes associated with behavioral performance together with genetic changes (Fig. 2). Recent findings from multiple studies in twins suggested that global and regional brain development and functions are actively controlled by genetics with a high heritability rate^121–125^. A review article published by Gallo et al., with an intensive focus on neural circuits and genetic variants implicated in developing ADHD symptoms, discusses how circuitry abnormalities relate to symptom presentation and treatment. Authors suggested that for unraveling, ADHD’s causality can be pinpointed by exploring the endophenotype fine-tuning in both basic and clinical environments by advanced studies in animal models, performing neuromodulation, and discoveries based on pharmaco-imaging^126^.

**Table 1:** Structural neuroimaging-genetics studies in ADHD.

Genes	Index variants	Groups	MRI	Imaging matrix	Results	References
*SLC6A3*	3’ UTR intron 8 VNTR haplotype	Three *SLC6A3* alleles (10/10 genotype, and the haplotypes 10–6 and 9–6)	sMRI	Bilateral striatal volumes (nucleus accumbens, CN, and putamen)	Adult ADHD 9–6 haplotype carriers ↑ 5.9% larger striatum volume relative to participants not carrying this haplotype.	Onnink et al.^130^
	3’ UTR VNTR	10R/10R carriers vs. 9R carriers	sMRI	Cingulate cortex thickness	10R/10R carriers: ↑ thickness in right cingulated gyrus and right BA 24.	Fernandez-Jaen et al.^131^
	3’ UTR VNTR	10R/10R carriers vs. 9R carriers	sMRI	PFC thickness	10R/10R carriers: ↓ cortical thickness in right BA 46 (lateral PFC).	Fernandez-Jaen et al.^129^
	3’ UTR VNTR	9R carriers vs. 10R/10R carriers, 4R/4R carriers vs. rest	sMRI	PFC GM and CN volume	SLC6A3 ADHD 10/10R carriers: ↓ CN volumes	Durston et al.^175^
	3’ UTR and intron 8 VNTR haplotype	10–6 haplotype carriers vs. non-10–6 haplotype carriers, 7R carriers vs. non-7R carriers	sMRI	Striatum, frontal cortex, and hippocampus volumes	SLC6A3 10–6 haplotype carriers: ↓ left striatal volume, irrespective of treatment.	Schweren et al.^134^
	3’ UTR VNTR	9R carriers vs. 10R/10R carriers	sMRI	CN volume	9R carriers: ↑ volumes of CN.	Shook et al.^176^
	3’ UTR VNTR, exon 3 VNTR, rs4680	9R carriers vs.10R/10R carriers; 4R/4R carriers vs.“rest	DTI	WM integrity, FA values	SLC6A3 9R carriers: no effect on WM integrity DRD4 4R/4R carriers: no effect on WM integrity	Hong et al.^140^
*COMT*	rs4680	Met carriers vs. Val/Val carriers	sMRI	Striatum, cerebellum, temporal lobe and IFG volume	ADHD Met carriers: ↓ GM volume. ADHD Val/Val: ↑ GM volume in right CN compared to ADHD Met carriers and HC.	Villemonteix et al.^135^
	rs4686	Met carriers vs. Val/Val carriers	sMRI	GM volume	ADHD Met carriers: ↓ GM volume in left putamen.	Shimada et al.^136^
	rs4680	Met carriers vs. Val/Val carriers	DTI	FA and RD values	ADHD Val/Val: ↓ FA and ↑ RD in the right cingulum (cingulated gyrus) compared to ADHD Met carriers and HC Val/Val.	Kabukcu Basay et al.^141^
	rs4680	Met carriers vs. Val/Val carriers	DTI	WM integrity, FA values	Met carriers: ↓ Network of WM connections linking 18” brain regions	Hong et al.^140^
*DRD4*	exon 3 VNTR	7R carriers vs. non-7R carriers	sMRI	Superior and middle frontal, anterior cingulate, and cerebellum cortices volumes	7R carriers: ↓ volumes of the superior frontal cortex and cerebellum cortex compared to non-carriers.	Monuteaux et al.^133^
	exon 3 VNTR	9R carriers vs. rest, 7R carriers vs. rest, S-allele carriers vs. rest	sMRI	Total GM, caudate, and putamen volume	Putamen volume, DRD4 7R carriers showed opposite age relations.	Richards et al.^153^
	exon 3 VNTR	7R carriers vs. non-7R carriers	sMRI	TBV, PFC, cerebellum, CN, and pallidum volume	No volumetric differences between 7R and non-7R carriers. No group × genotype interactions.	Castellanos et al.^177^
	exon 3 VNTR	9R carriers vs. 10R/10R carriers, 4R/4R carriers vs. rest	sMRI	PFC GM and CN volume	DRD4 unaffected siblings 7R carriers: ↑ prefrontal GM volume.	Durston et al.^177^
	exon 3 VNTR	10–6 haplotype carriers vs. non-10–6 haplotype carriers, 7R carriers vs. non-7R carriers	sMRI	Striatum, frontal cortex, and hippocampus volumes	DRD4 7R carriers: frontal cortex volume is associated with stimulant treatment at a younger age.	Schweren et al.^134^
	exon 3 VNTR	9R carriers vs.10R/10R carriers; 4R/4R carriers vs. rest	DTI	WM integrity, FA values	DRD4 4R/4R carriers: no effect on WM integrity	Hong et al.^140^
*SLC6A4*	5-HTTLPR	S carriers vs. LL carriers	sMRI	GM volume	S carriers: stress exposure is associated with ↓ GM volume in the precentral gyrus, middle and superior frontal gyri, frontal pole, and cingulated gyrus. Association of GxE interaction with ADHD symptom count was mediated by GM volume in frontal pole and anterior cingulated gyrus only.	van der Meer et al.^178^
	5-HTTLPR	S-allele carriers vs. rest	sMRI	GM volume	↑ positive relation between stress exposure and ADHD severity; Interactions were reflected in GM volume of cerebellum, parahippocampal gyrus, intracalcarine cortex,and angular gyrus.	van der Meer et al.^179^
	5-HTTLPR	S-allele carriers vs. rest	sMRI	Total GM, caudate, and putamen volume	For total GM, differential age effects were found for SLC6A4 L/L carriers, depending on the amount of positive peer affiliation.	Richards et al.^153^
*SLC6A3*	*SLC6A3* 3’ UTR VNTR; HTTLPR	9R carriers vs. rest, 7R carriers vs. rest, S-allele carriers vs. rest	sMRI	Total GM, caudate, and putamen volume	For total GM, differential age effects were found for SLC6A3 9R- and SLC6A4 L/L carriers, depending on the amount of positive peer affiliation.	Richards et al.^153^
	3’ UTR VNTR, exon 3 VNTR, rs4532	9R carriers vs. 10R/10R carriers, 7R carriers vs. non-7R carriers, C-allele carriers vs. non-C-allele carriers	sMRI	Cortical thickness	SLC6A3 9R carriers: No effect. DRD4 7R carriers: thinner right orbitofrontal/inferior prefrontal and posterior parietal cortex. ADHD 7R carriers: distinct trajectory of cortical development; normalization of the right parietal cortical region.	Shaw et al.^132^
*SLC6A2*	rs4532; rs265981, rs998424, rs3785157	2 and 3 genotype groups per SNP	sMRI	TCV, volumes of total GM and WM, CN, cerebellum, frontal, temporal, parietal lobes	NET1 SNPs: no genotype effects on GM or WM volume and no group × genotype interactions.	Bobb et al.^180^
*DRD1*	3’ UTR VNTR, exon 3 VNTR, rs4532	9R carriers vs. 10R/10R carriers, 7R carriers vs. non-7R carriers, C-allele carriers vs. non-C-allele carriers	sMRI	Cortical thickness	DRD1: no effect of genotype on the clinical outcome or cortical development.	Shaw et al.^132^
	rs4532; rs265981, rs998424, rs3785157	2 and 3 genotype groups per SNP	sMRI	TCV, volumes of total GM and WM, CN, cerebellum, frontal, temporal, parietal lobes	DRD1 and NET1 SNPs: No genotype effects on GM or WM volume and no group × genotype interactions.	Bobb et al.^180^
*NOS1*	exon 1f-VNTR	SS carriers vs. SL/LL carriers	DTI	WM integrity, FA and MD values	Female SS carriers: ↑ MD in right parietal WM tracts. Males: no difference between genotype groups. No genotype × diagnostic group interaction.	van Ewijk et al.^181^
*ADRA2A*	rs1800544, rs553668	C-allele carriers vs. GG carriers, T-allele carriers vs. CC carriers	DTI	White matter integrity, FA values	rs1800544 C-allele carriers: ↓ FA in the right postcentral gyrus. rs553668 T-allele carriers: ↓FA in the right middle frontal cortex.	Park et al.^182^
*NR3C1*	5-HTTLPR, rs6189, rs6198	S-allele carriers vs. rest; NR3C1 risk haplotype carriers (rs6189G and rs6198G) vs. rest	sMRI	GM volume	NR3C1 risk haplotype carriers: ↑ positive relation between stress exposure and ADHD severity	van der Meer et al.^179^

### Genes associated with the anatomical brain changes

**Fig. 8:**
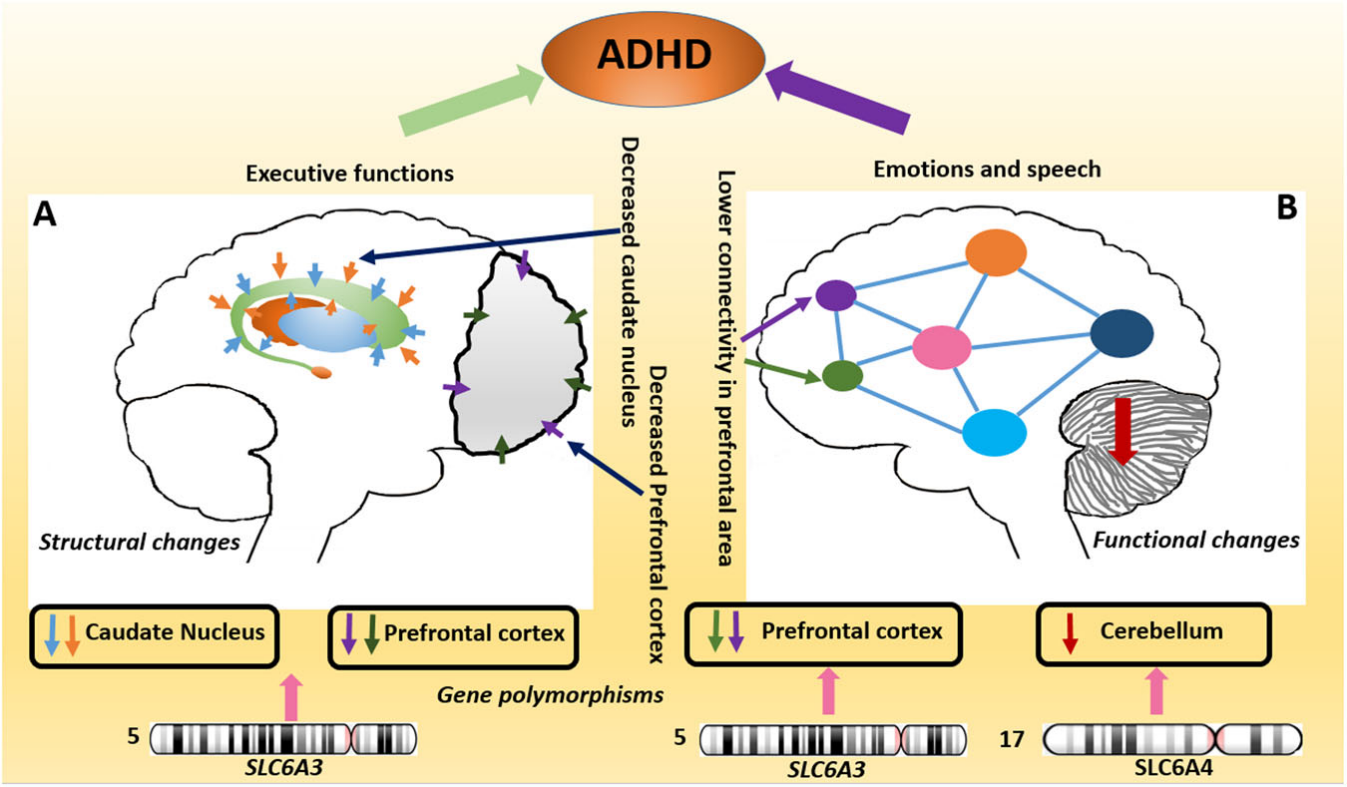
The figure shows structural and functional brain changes associated with gene polymorphisms in patients with ADHD. **A** *SLC6A3* polymorphisms are associated with lower caudate nucleus volume and prefrontal cortex in patients with ADHD. **B** *SLC6A3* and *SLC6A4* polymorphisms associated with lower functional activity in the prefrontal cortex and cerebellum in the brain of ADHD (Figure inspired from the manuscript by Tripp G, et al., 2009^188^).

Structural MRI offers the opportunity to understand the effect of gene polymorphisms on anatomical changes in various neurological and psychiatric disorders, including ADHD. Smaller global and regional brain volumes and subcortical structures together with multiple gene polymorphisms are reported as the risk factors for ADHD (Table 1), and one such example of gene polymorphism is Dopamine transporter solute carrier family 6 member 3 (*SLC6A3);* previously known as *DAT1* gene which encodes the transmembrane proteins involved in reuptake of dopamine from the synapse (Fig. 8).

A study performed on children with ADHD observed that *SLC6A3* haplotype is associated with decreased gray matter volume in multiple brain regions, including the left superior occipital region, cuneus, precuneus, and the right angular areas. It was suggested that the abnormalities in these brain regions might be responsible for the impairment of visual memory in ADHD children^127^. Long-term treatment with psychostimulant drugs such as methylphenidate (MPH), which acts by inhibiting reuptake of dopamine and norepinephrine or Atomoxetine helps to increase gray matter in the prefrontal and occipital areas of ADHD children having 10/10-repeat allele with a variable number tandem repeat of 40 bp of the *SLC6A3* genotype associated with ADHD compared to TD children^128^.

A study based on cortical thickness measurements in patients with ADHD observed that ADHD patients carrying the *SLC6A3* gene with two copies of the 10R allele showed lower cortical thickness in the right lateral prefrontal cortex compared to one or the absence of the 10R allele^129^. While investigating the effect of three *SLC6A3* alleles (10/10 genotype, and the haplotypes 10–6 and 9–6) on striatum volume in ADHD patients, another volumetric-based study reported that carriers of the *SLC6A3* haplotype 9–6 had around 6% bigger striatum volume than non-carriers^130^. In another seminal study, Fernandez-Jaen A et al. found that ADHD children homozygous for *SLC6A3* with a 10-repeat allele had significantly higher cortical thicknesses in the right ventral and cingulate gyrus relative to 9-repeat carriers. The authors also suggested that the presence of the 10-repeat allele in ADHD influences the cortical thickness of the cingulate gyrus^131^.

An age-dependent study observed an association of *DRD4* 7-repeat allele with lower cortical thickness in the right orbitofrontal/inferior prefrontal and posterior parietal brain sites in children with ADHD^132^. Another study observed that patients with ADHD having a 7-repeat allele of the *DRD4* gene showed a lower volume of the superior frontal cortex and cerebellum cortex than ADHD patients without the *DRD4* 7-repeat allele. It was further suggested that volume changes in the brain of ADHD might indicate an intermediate morphological phenotype between the DRD4 genotype and the expression of the clinical characteristics of ADHD^133^. A structural MRI study has been performed to examine the effect of MPH treatment on brain structures in ADHD patients carrying the *DRD4* 7R allele. After treatment, increased volume of the frontal cortex and left hippocampal was observed in younger patients with ADHD, suggesting that younger patients with ADHD carrying *DRD4* genotype are more sensitive to cortical remodeling after stimulant treatment^134^.

A voxel-based morphometry study was performed to explore the impact of *COMT* Val158Met polymorphism on grey matter in children with ADHD, and it was found that the presence of Met158-allele is associated with lower gray matter volume in the inferior frontal gyrus. However, children with ADHD homozygotes for the Val158-allele showed higher gray matter in the caudate nucleus when compared with TD children^135^. The *COMT* gene is involved in the deactivation of neurotransmitters such as dopamine, epinephrine, and norepinephrine and regulates the function of the enzymes involved in their synthesis. A study performed in ADHD to look for the differential effect of *COMT* on the brain of different ethnic groups observed that *COMT* polymorphism resulted in lower gray matter volume in the left striatum in ADHD children compared to healthy children. The *COMT* Met carrier ADHD children showed lower striatal gray matter volume than *COMT* Val/Val-genotype ADHD children. Among Caucasian children with ADHD, striatal gray matter volume alterations are correlated with the *COMT* Val-homozygotes. In contrast, in Japanese children with ADHD, striatal gray matter volume alterations are associated with the *COMT* met allele. Such findings suggest ethnic differences in the genetic effects of *COMT* on brain changes in ADHD patients^136^.

The solute carrier family 6 member 4 (*SLC6A4)* gene encodes the integral membrane protein that regulates the serotonin transport from synaptic spaces into presynaptic neurons and polymorphism in this gene is associated with a higher risk of ADHD. It has been shown that *SLC6A4* methylation is associated with lower cortical thickness in the right occipitotemporal region in children with ADHD^137^.

Polymorphism of gene coding for a synaptosomal associated protein (*SNAP25*) was associated with altered gray matter volume in ADHD patients^138^. The *DBH* gene involved in the synthesis of an enzyme dopamine beta-hydroxylase, responsible for the conversion of dopamine to norepinephrine, is presumed to play an essential role in the autonomic nervous system. A surface measurement-based study showed that *DBH* gene polymorphism is associated with larger left insula surface area in ADHD children with G carriers than AA homozygotes^139^.

### Genes associated with white matter changes

The white matter in the human brain is highly heritable and plays an important determinant of interindividual differences in brain functions such as cognition and can contribute to neuropsychiatric disorders. The structural composition and architecture of the brain, such as white matter connectivity and gene polymorphism are related to ADHD. There are limited studies available to assess the association of changes in white matter structures with gene polymorphisms in ADHD (Table 1). Hong SB et al. used DTI to evaluate the white matter connectivity in ADHD patients with *COMT* Val-homozygous and *COMT* Met carriers. They found decreased white matter connections in ADHD patients with *COMT* Met carriers than those of *COMT* Val-homozygous^140^. Another DTI study found that children with ADHD bearing *COMT* Val homozygote exhibited significantly reduced fractional anisotropy and increased radial diffusivity in the right cingulate gyrus relative to those of *COMT* Met carriers and healthy controls with homozygote *COMT* Val genotype profile. Also, ADHD children with *COMT* Met carriers had increased fractional anisotropy and axial diffusivity in the left uncinate fasciculus and decreased radial diffusivity in left posterior corona radiata and posterior thalamic radiation compared to ADHD children with homozygote *COMT* Val. These findings suggest that *COMT* polymorphism influenced the development of white matter in ADHD infants carrying Val homozygote^141^. While evaluating the association of the *DRD4-5*-repeat allele with mean diffusivity, investigators of another study observed a significantly increased mean diffusivity in the gray and white matter areas with the expression of the *DRD4* 5-repeat allele, which could be an increased risk factor for developing ADHD in children^142^.

### Genes associated with functional brain changes

Genetic variations, especially gene polymorphisms of the various genes such as *DRD4, SLC6A3, DRD1*, neuroepithelial cell transforming 1 (*NET1)* etc. have been shown to influence the functional brain activity in patients with ADHD (Table 2)^143–148^. An rs-fMRI study observed that the absence of a 2-repeat allele of the *DRD4* gene in ADHD children is associated with hyperconnectivity in the default mode network and sensorimotor network and hypoconnectivity in the executive control network compared to ADHD children who showed the presence of 2-repeat allele. This suggests that polymorphism of the *DRD4* 2-repeat allele influences the network connectivity associated with inattention activity^149^. Another study from the same group on the effect of *DRD4* (4R/4 R vs. 2R) gene polymorphism on ReHo and functional connectivity in ADHD patients found that the presence of the *DRD4* 2R allele had both increased and decreased ReHo bilaterally in the cerebellum and the left angular gyrus, respectively. Patients with the *DRD4* 2R allele also showed lower functional connectivity to the left angular gyrus in the left striatum, right inferior frontal gyrus, bilateral lobes of the cerebellum, and increased functional connectivity in the left superior frontal gyrus, medial frontal gyrus, and rectus gyrus. Based on these findings, the authors suggested that *DRD4* polymorphisms are associated with localized brain activity and specific functional connections^150^. Another study based on rs-fMRI observed decreased ReHo in the left superior occipital gyrus, cuneus, and precuneus in ADHD patients having *SLC6A3* polymorphism *SLC6A3* rs27048 (C)/rs429699 (T) haplotype and without the CT haplotype). Significant interactions of the ADHD disorder status (diagnosis) and CT haplotype with decreased ReHo were observed in the right postcentral gyrus^127^.

**Table 2:** Functional neuroimaging-genetics studies in ADHD.

Genes	Index variants	Groups	MRI	Imaging matrix	Results	References
*SLC6A3*	9–6 *SLC6A3* haplotype	9–6 haplotype carriers vs. non 9–6 haplotype carriers	fMRI	Rewarded and non‐rewarded trials	Bayesian Constraint-based Causal Discovery (BCCD) algorithm confirmed that there is no direct link between *SLC6A3* genetic variability and brain activation, but suggested an indirect link mediated through inattention symptoms and diagnostic status of ADHD.	Sokolova et al.^146^
	9‐6 *SLC6A3* haplotype	*SLC6A3* 10–6 dosage (2 copies vs. <2 copies)	fMRI	VS and CN activity during reward-predicting cues	ADHD: activation in CN ↓ as a number of copies ↑, but in the control group reverse was found.	Paloyelis et al.^143^
	3’ UTR VNTR	9R carriers vs. 10R/10R carriers	fMRI	Working memory task	9R carriers: ↓ left medial PFC activation compared to 10R/10R carriers. “Group × genotype interaction showed that 10R/10R-ADHD” “patients had ↑ activity in pre-SMA/dorsal ACC compared” to HC.	Brown et al.^183^
	3’ UTR VNTR	10R/10R carriers vs. 9R carriers	fMRI	Go/No-Go task	10R/10R carriers: ↑ activity in frontal, medial, and parietal regions during response inhibition compared to 9R carriers; ↓error response in the parahippocampal gyrus.	Braet et al.^184^
	3’ UTR VNTR	10R/10R carriers vs. 9R carriers	fMRI	Go/No-Go task	10R/10R carriers: ↑ activity in left striatum, right dorsal premotor cortex, and temporoparietal cortical junction compared to 9R carriers.	Bedard et al.^144^
	3’ UTR VNTR	9R carriers vs. 10R/10R carriers	fMRI	Go/No-Go paradigm	9R carriers: ↑ activity in CN and ↓ in cerebellar vermis compared to 10R/10R carriers. Group × genotype interaction: effect in CN is observed in ADHD and unaffected siblings, but not HC.	Durston et al.^185^
	3’ UTR VNTR	10R/10R carriers vs. 9R carriers	fMRI	Multi-source interference task	9R carriers: ↓ activity in dorsal ACC compared to 10R/10R carriers.	Brown et al.^161^
	3’ UTR and intron 8 VNTR haplotype	9–6 haplotype carriers vs. non 9–6 haplotype carriers	fMRI	Striatal activity during reward anticipation task	No differences in striatal activity compared with non 9–6 haplotype carriers nor 9R- and 10R/10R carriers.	Hoogman et al.^148^
	3’ UTR and intron 8 VNTR haplotype, rs37020, rs460000, rs4680	10–6 haplotype carriers vs. non-10–6 haplotype- carriers; rs37020	fMRI	Stop signal task	No genotype × ADHD interaction effects. SLC6A3 10–6 “haplotype-homozygotes: ↑ activity related to successful” stop-trials in pre-supplementary motor “areas, ↓ activity in superior frontal and temporal pole” areas. “rs37020 AA carriers: ↓ activity during failed stop-trials in IFG,” pre-supplementary motor areas, and postcentral gyrus.	van Rooij et al.^158^
*COMT*	rs4680	Met carriers vs. Val/Val carriers	rs-fMRI	Crus I/II in the cerebellum	Met-carriers exhibiting significantly lower functional connectivity than the Val/Val genotype.	Mizuno et al.^160^
*SLC6A4*	5-HTTLPR	3 genotype groups per variant	fMRI	Stop signal task	SLC6A4 SS-genotype group: ↓ activation in frontal nodes and “↑ activation in posterior nodes. No associations between SLC6A4 and HTR1B variants and ADHD or ADHD-related neural activation.	van Rooij et al.^159^
*NOS1*	exon 1f-VNTR	SS carriers vs. SL/LL carriers	fMRI	Reward anticipation task/modified MID task	SS carriers: ↑ activity in VS. No group × genotype interactions.	Hoogman et al.^186^
*MAOA*	rs1137070	TT carriers vs. CC carriers	fMRI	Working memory task	ADHD TT carriers: ↑ activation in the left inferior frontal lobe, pars opercularis.	Ko et al.^147^
*HTR1B*	5-HTTLPR, rs6296	3 genotype groups per variant	fMRI	Stop signal task	SLC6A4 SS-genotype group: ↓ activation in frontal nodes and “↑ activation in posterior nodes. HTR1B genotype: associated” with differential activation in anterior cingulate, occipital, inferior temporal, and cerebellar regions during successful stop trials. No associations between SLC6A4 and HTR1B variants and ADHD or ADHD-related neural activation.	van Rooij et al.^159^

The *N*-methyl-d-aspartate (NMDA) and dopamine receptor genes are found to have significant effects on functional connectivity in ADHD. A recent study investigated the effects of *NMDA* receptor gene glutamate ionotropic receptor NMDA type subunit 2B (*GRIN2B)* and dopamine receptor gene (*DRD4)* variants on ReHo in the ADHD group and healthy controls by using rs-fMRI. They found that the ADHD group with *GRIN2B* TC/TT genotype showed lower static and dynamic ReHo in the left superior parietal surface than the healthy controls. In contrast, the ADHD group with *DRD4* variable number tandem repeat (VNTR)2 polymorphism showed lower dynamic ReHo in the right superior parietal surface. Considering the role of the superior parietal region in the selective attention process, decreased static and dynamic ReHo in the ADHD group in the superior parietal region may lead to worse performance outcomes during active states and may reduce the ability to respond to attention-based tasks in ADHD. Based on these findings, this study concluded that alterations in dopaminergic and glutamatergic systems contribute to impaired local functional connectivity leading to attention deficits in ADHD patients^151^.

A stop-signal task-based fMRI study was performed to measure response inhibition task in individuals carrying ADHD risk alleles of the *DRD4* and *SLC6A3* genes. The authors found that carriers of *DRD4* 7-repeat allele showed reduced activation in the superior frontal and middle gyrus during successful response inhibition and reduced activation in the supramarginal gyrus and parietal lobule during failed response inhibition. In contrast, *SLC6A3* risk variants showed lower cerebellar activity during failed trials of successful response inhibition^152^. Another study explored to find interactions between variants in candidate plasticity genes (*SLC6A3, SLC6A4*, and *DRD4*) and social environments (maternal expressed emotion and peer affiliation). The study showed that exposure to high positive peer affiliation was associated with the least reward speeding in serotonin-transporter-linked polymorphic region *(HTTLPR)* short allele carriers, while exposure to low positive peer affiliation or low maternal warmth was associated with the most reward speeding in *HTTLPR* short allele carriers. On the other hand, *SLC6A3* 10-repeat homozygotes carriers displayed the most extended reaction times when exposed to low maternal warmth. In contrast, *DRD4* 7-repeat carriers showed high neural activation when exposed to little maternal warmth and vice versa. Taken together, these findings emphasize the relevance of supportive social environments in sensitivity rewards and task performance with specific genotypes has differential environmental impacts^153^.

ADHD heterogeneity is one of the major issues to be found in a study evaluating the neurobiological pinning of a new ADHD phenotype known as ADHD restrictive inattentive (ADHD-RI) and comparing ADHD-RI, ADHD inattentive, combined ADHD and TD individuals using genetic data involving dopamine transporters and receptor gene polymorphisms (*SLC6A3* and DRD4) and by performing fMRI (go/no task). The study found that children with ADHD-RI showed reduced psychomotor speeds and higher activation of temporo-occipital regions during the Go/No-Go task as compared to TD individuals. In addition, ADHD-RI children had a higher presence of *DRD4*-7-repeat allele^154^. Another study employed DTI and N-back fMRI paradigms to investigate the effect of the *DRD4*-5-repeat allele on microstructural properties and functional connectivity in the brain in a healthy Asian population, mostly comprising of adolescent individuals. The study found that the presence of a 5-repeat allele was associated with poor processing speed performance, increased impulsivity, and reduced tendency to maintain attentional focus, suggesting that the presence of the 5-repeat allele of the *DRD4* gene might contribute to the risk of developing ADHD^142^. A study conducted by Gilsbach S et al. investigated the effect of DRD4-7-repeat allele in a healthy population of children and adolescents using combined stimulus-response incompatibility task (IC) and time-discrimination task (TT). The study showed that the DRD4-7-repeat carriers demonstrated reduced neural activation of the middle and frontal gyrus in IC and reduced cerebellum activation in TT. Also, the *DRD4*-7 carriers showed reduced coupling between frontal brain regions compared to 7-repeat non-carriers^155^. An fMRI study investigated the effect of *SLC6A3* VNTR polymorphism on the brain’s activity in a working memory task in ADHD and TD children. Authors observed that working memory-related activation was more significant in 9R carriers in ADHD subjects and only 10R homozygotes showed higher working memory-related activation than 9R carriers in multiple brain sites, including the parietal, temporal lobes, ventral visual cortex, orbitofrontal gyrus, and the head of the caudate nucleus in ADHD children. The findings suggest that the presence of *SLC6A3* polymorphism can significantly influence the working memory in ADHD children^156^. A study performed a verbal n-back task in two fMRI runs to see the effect of one (9/10) copy of the 10-repeat allele of the *SLC6A3* genotype in TD children. The study observed that 9/10 carriers showed more activation in frontal–striatal–parietal regions than 10/10 carriers in high load run. On the contrary, subthalamic nuclei tended to be more activated in 10/10 carriers under low load, which showed that *SLC6A3* 10R homozygosity is associated with reduced performance in higher demanding working memory tasks^157^. Another task-based study performed Go and No-Go paradigm to assess the impact of *SLC6A3* 3’ UTR genotype polymorphisms on brain activation in unmedicated ADHD youth and children. Youth with the *SLC6A3* 3’ UTR 10R/10R genotype showed higher activation in the left striatum, right dorsal premotor cortex, and bilaterally in the temporoparietal cortical junction as compared to the ADHD individuals who were heterozygous for the *SLC6A3* 3′ UTR 9R allele. These findings provide preliminary evidence that neural activity related to inhibitory control may differ as a function of *SLC6A3* 3′ UTR genotype in youth with ADHD^144^.

In patients with ADHD, dopamine and serotonin-related genes play an essential role in the neurobiological response of inhibition and large-scale neural activation changes. A study observed large-scale changes in the response inhibition networks’ neural activation in prefrontal, parietal, and subcortical regions in relation to *SLC6A3* and *COMT* polymorphisms in ADHD patients. A similar study showed large-scale differences in neural activation in the frontal and parietal regions of the response inhibition network between different variants of *HTR1B* and solute carrier family 6 member 4 (*SLC6A4)*, previously known as *5HTT* genes in ADHD patients^158,159^.

A study examined whether *COMT* polymorphism is associated with the alteration of cortico-cerebellar executive function in ADHD children using rs-fMRI. The study showed that ADHD *COMT* Met carriers exhibited decreased functional connectivity of right Crus I/II with the left dorsolateral prefrontal cortex compared to the ADHD children with Val/Val genotype^160^. Another study by Brown AB et al. showed that ADHD subjects homozygous for the *SLC6A3* 10R allele showed significant hypoactivation in the left dorsal anterior cingulate cortex, lateral prefrontal cortex, and cerebellar vermis as compared to *SLC6A3* 9R carriers. The *SLC6A3* 9R carriers showed greater activation in the left dorsal anterior cingulate cortex and cerebellar vermis and right lateral prefrontal cortex compared to the *SLC6A3* 10R carriers^161^. A study investigating the association between synaptosomal associated protein (*SNAP25*) rs3746544 polymorphism and functional connectivity density (FCD) in male children with ADHD found that rs3746544 TT homozygous carriers showed decreased local functional connectivity hubs in the anterior cingulate cortex and dorsal lateral prefrontal cortex as compared to rs3746544 G-allele carriers, suggesting that the *SNAP25* polymorphism is linked with ADHD^162^. An additional study investigating the effect of *MAOA* genotype using stop signal fMRI task in adolescent boys and girls found that MAOA was correlated with ADHD symptoms and subsequently, single-nucleotide polymorphism (SNP) rs12843268 “A” hemizygotes lowered MAOA levels and reduced ventral striatal BOLD response during monetary incentive delay task. Whereas in “G” hemizygotes of SNP rs12843268 associated with higher MAOA levels and frontal gyrus and ventral striatal hyperactivation during monetary incentive delay task, and frontal gyrus hypoactivation during the stop signal task^163^. Another fMRI study conducted working memory tasks in adults with ADHD to investigate the effect of *MAOA* polymorphisms on working memory, distraction, and dual-tasking. The authors found increased activation for working memory in the lower bilateral frontal lobe and pars opercularis, and increased activation in the lingual gyrus in response to dual-tasking^147^.

### Pharmacogenomics and brain metabolism

The brain metabolites or neurotransmitters’ levels are strictly controlled and regulated by both genetic and epigenetic factors. Gene abnormalities or polymorphisms can lead to changes in brain metabolite levels in ADHD via alterations of the cortico–striato–thalamic–cortical networks. Using ^1^H MRS, a study investigated the relationship between *SLC6A3* gene polymorphisms and brain metabolite responses in patients with ADHD following administration of MPH drug. Interestingly, no significant differences in NAA, Cr, and Cho levels were observed before and after drug administration. In ADHD individuals with *SLC6A3* 10R genotype, only high levels of Cr were identified in the cerebellum after MPH administration^164^. The increase in Cr levels (hypermetabolic state) after MPH administration might have been due to the normalization of CBF and glucose metabolism following psychostimulant therapy in ADHD. Similarly, in ADHD adults with *SNAP25* gene polymorphism, another MRS study evaluated brain metabolite responses to MPH treatment. The study found high levels of NAA in adults with *SNAP25* DdeI (rs1051312) and *SNAP-25* MnlI (rs3746544) polymorphisms in the anterior cingulate cortex region after MPH treatment, suggesting that NAA levels in ADHD may be influenced by changes related to MPH^165^. Another MRS study examined changes in neurometabolite levels in ADHD adults with synapsin III *(SYN3)* gene polymorphisms in response to MPH. The study found higher levels of Cho in the striatum of ADHD subjects with synapsin III rs133945 polymorphism and higher NAA level in ADHD subjects with synapsin III rs133945 polymorphism^166^. A similar MRS study was conducted to determine the effect of treatment with MPH on brain metabolite levels in ADHD subjects with *COMT* gene polymorphism. Increased levels of NAA were reported in the anterior cingulate cortex and prefrontal dorsolateral cortex regions in the Val/Val and Val/Met genotype (rs4680) carriers and elevated levels of Cho were reported in the Val/Val and Val/Met genotype carriers striatum after treatment with MPH suggesting that MPH had a positive impact on impaired neuronal function and activity^167^. We believe that the integration of pharmacogenomics and metabolomics in future studies may open up new horizons in the diagnosis and treatment of ADHD.

## Future perspective

Accurate and specific diagnosis or profiling of ADHD symptoms remains a significant challenge in the pediatric cohort^168^. Notably, it is difficult to distinguish whether the symptoms are signs of ADHD or merely a sign of a young active child or symptoms of other neurodevelopmental or neuropsychiatric disorders^169–171^. The fundamental challenge in diagnosing ADHD is that the symptoms of ADHD are distributed in the general population, with a wide range of factors contributing to the etiology^172^. Since ADHD comprises different subtypes (inattention, hyperactive, and combined) and comorbidity with psychiatric conditions, a joint research strategy on ADHD and other neuropsychiatric disorders is required to define the basis of these phenotypes. The use of targeted next-generation sequencing for coding and noncoding regions can identify different genes and pathways involved in ADHD, paving the way for the development of enhanced diagnostic tools with improved treatment outcomes^173^. The advancement in neuroimaging and molecular genomics offers the opportunity to examine genetic variations on imaging-derived phenotypes in ADHD and the development of polygenic risk scores to predict the risk of developing ADHD and improve the diagnosis and tailored treatments. The epigenetic pathways involved in ADHD must be investigated and how they interact with genetic factors or risks involved in ADHD. Because of the broad-ranging consequences of the heterogenic nature of ADHD, it may be challenging to unravel the entire genetic profile of ADHD. However, it still appears to be achieved through genomic technology progressions and advanced neuroimaging. There is also a need to link the increasing evidence of genetic anomalies in ADHD with measures of brain dysfunction in longitudinal studies to determine whether the brain abnormalities change throughout the life cycle. It would be also helpful to conduct studies that use identical measures to assess neurobiological continuity in children and adults with ADHD. Besides clinical interventions, pharmaceutical medications that include psychostimulants are the most widely recognized medicines used to treat ADHD symptoms but have been found to be of limited utility due to the heterogeneous nature of ADHD disorder^174^.

## Conclusion

The ADHD neurobiology is intricate and involves multiple neural pathways, but dopamine, noradrenaline, and serotonin are the critical neurotransmitters highlighted in ADHD pathogenesis. The combined efforts from psychologists, psychiatrists, geneticists, and neuroscientists have resulted in an improved understanding of ADHD etiology. Given the multifaceted symptomatology and multifactorial origin of ADHD, significant research efforts have been made over the past years to explore ADHD-related genetic and neural modifications. Notwithstanding the involvement of candidate genes and neurotransmitter systems in ADHD, genome-wide correlations between ADHD symptoms and individual genetic variants have yet to be established. Therefore, their contributions to our understanding of the etiology of ADHD are limited. Larger-scale, multicenter neuroimaging genetic approaches are now in progress, however, representing one hopeful avenue to translate this polygenic disorder’s genetic architecture. Besides, there has been considerable progress in finding vital brain circuits and regions whose structure, function, and connectivity are impaired in ADHD. One of the biggest challenges yet to be faced is developing causal relationships between these neural fluctuations and the disorder. Improved neuroimaging methods combined with experimental manipulations such as advanced neuromodulation and pharmacological approaches would probably require a deeper understanding of neural circuits and their functions. Also, relying on well-characterized animal models and the latest technologies, such as in vivo optogenetics, could allow selective manipulation of the neural circuitry involved during ADHD-related tasks. A critical future direction for ADHD research is to couple human and animal neuroimaging genetic studies to explore how the risk genes associated with ADHD neurobiology affect the brain changes in knock-out rodent models. This will help to identify the abnormal biological pathways involved in ADHD pathophysiology.

The intermediate or endophenotype approach allows mapping the effects of individual risk genes on neurobiological parameters, such as brain structure, chemistry, and ultimately function. In addition, the combination of neuroimaging-related endophenotypes with genetic networks is now seen as an explanatory combinatorial model for understanding detailed ADHD pathogenesis including the generation of polygenic risk scores. The combination of neuroimaging, psychiatric genetics, and behavioral genetics will not only contribute to the diagnosis of ADHD but can also be a useful tool for personalized medicine. In the years to come, we will gain a more comprehensive understanding of ADHD, thereby allowing for new medications that are more successful than those currently in use.

## References

[1] Polanczyk G, Rohde LA (2007). Epidemiology of attention-deficit/hyperactivity disorder across the lifespan. Curr. Opin. Psychiatry.

[2] Simon V, Czobor P, Balint S, Meszaros A, Bitter I (2009). Prevalence and correlates of adult attention-deficit hyperactivity disorder: meta-analysis. Br. J. Psychiatry.: J. Ment. Sci..

[3] Nigg JT (2013). Attention deficits and hyperactivity-impulsivity: what have we learned, what next?. Dev. Psychopathol..

[4] Wilens TE, Spencer TJ (2010). Understanding attention-deficit/hyperactivity disorder from childhood to adulthood. Postgrad. Med..

[5] Buitelaar JK (2012). Long-term efficacy and safety outcomes with OROS-MPH in adults with ADHD. Int. J. Neuropsychopharmacol..

[6] Haavik J, Halmoy A, Lundervold AJ, Fasmer OB (2010). Clinical assessment and diagnosis of adults with attention-deficit/hyperactivity disorder. Expert Rev. Neurotherapeut..

[7] Scott, J. G., Mihalopoulos, C., Erskine, H. E., Roberts, J. & Rahman, A. Childhood mental and developmental disorders. in *Mental, Neurological, and Substance Use Disorders: Disease Control Priorities*, *Third Edition* (eds Patel, V. et al.) Vol. 4, 145–161 (2016).

[8] Yurtbasi P (2018). Comparison of neurological and cognitive deficits in children with ADHD and anxiety disorders. J. Atten. Disord..

[9] Quintero J (2018). Health care and societal costs of the management of children and adolescents with attention-deficit/hyperactivity disorder in Spain: a descriptive analysis. BMC Psychiatry.

[10] Martinez-Raga, J., Ferreros, A., Knecht, C., de Alvaro, R. & Carabal, E. Attention-deficit hyperactivity disorder medication use: factors involved in prescribing, safety aspects and outcomes. *Ther. Adv. Drug Saf.* **8**, 87–99 (2017). 10.1177/2042098616679636 PMC5367662 28382197

[11] Ogundele MO (2018). Behavioural and emotional disorders in childhood: a brief overview for paediatricians. World J. Clin. Pediatr..

[12] Posner J, Polanczyk GV, Sonuga-Barke E (2020). Attention-deficit hyperactivity disorder. Lancet.

[13] Cortese S, Castellanos FX (2012). Neuroimaging of attention-deficit/hyperactivity disorder: current neuroscience-informed perspectives for clinicians. Curr. Psychiatry Rep..

[14] Klein M (2017). Brain imaging genetics in ADHD and beyond—mapping pathways from gene to disorder at different levels of complexity. Neurosci. Biobehav. Rev..

[15] Weyandt L, Swentosky A, Gudmundsdottir BG (2013). Neuroimaging and ADHD: fMRI, PET, DTI findings, and methodological limitations. Dev. Neuropsychol..

[16] Samea F (2019). Brain alterations in children/adolescents with ADHD revisited: a neuroimaging meta-analysis of 96 structural and functional studies. Neurosci. Biobehav. Rev..

[17] Mueller A, Hong DS, Shepard S, Moore T (2017). Linking ADHD to the neural circuitry of attention. Trends Cogn. Sci..

[18] Stevens MC, Pearlson GD, Calhoun VD, Bessette KL (2018). Functional neuroimaging evidence for distinct neurobiological pathways in attention-deficit/hyperactivity disorder. Biol. Psychiatry.: Cogn. Neurosci. Neuroimaging.

[19] Faraone SV, Mick E (2010). Molecular genetics of attention deficit hyperactivity disorder. Psychiatr. Clin. North Am..

[20] Larsson H, Chang Z, D’Onofrio BM, Lichtenstein P (2014). The heritability of clinically diagnosed attention deficit hyperactivity disorder across the lifespan. Psychol. Med..

[21] Klein M (2017). Brain imaging genetics in ADHD and beyond—mapping pathways from gene to disorder at different levels of complexity. Neurosci. Biobehav. Rev..

[22] Williams NM (2012). Genome-wide analysis of copy number variants in attention deficit hyperactivity disorder: the role of rare variants and duplications at 15q13.3. Am. J. Psychiatry.

[23] Jarick I (2014). Genome-wide analysis of rare copy number variations reveals PARK2 as a candidate gene for attention-deficit/hyperactivity disorder. Mol. Psychiatry.

[24] Acosta MT (2016). ADGRL3 (LPHN3) variants are associated with a refined phenotype of ADHD in the MTA study. Mol. Genet. Genom. Med..

[25] Silva JP (2011). Latrophilin 1 and its endogenous ligand Lasso/teneurin-2 form a high-affinity transsynaptic receptor pair with signaling capabilities. Proc. Natl Acad. Sci. USA.

[26] Arcos-Burgos M (2010). A common variant of the latrophilin 3 gene, LPHN3, confers susceptibility to ADHD and predicts effectiveness of stimulant medication. Mol. Psychiatry.

[27] Khadka S (2016). Multivariate imaging genetics study of MRI gray matter volume and SNPs reveals biological pathways correlated with brain structural differences in attention deficit hyperactivity disorder. Front. Psychiatry.

[28] Kebir O, Tabbane K, Sengupta S, Joober R (2009). Candidate genes and neuropsychological phenotypes in children with ADHD: review of association studies. J. Psychiatry Neurosci..

[29] Demontis D (2019). Discovery of the first genome-wide significant risk loci for attention deficit/hyperactivity disorder. Nat. Genet..

[30] Cortese S (2012). The neurobiology and genetics of Attention-Deficit/Hyperactivity Disorder (ADHD): what every clinician should know. Eur. J. Paediatr. Neurol..

[31] Greven CU (2015). Developmentally stable whole-brain volume reductions and developmentally sensitive caudate and putamen volume alterations in those with attention-deficit/hyperactivity disorder and their unaffected siblings. JAMA Psychiatry.

[32] Shaw P (2007). Attention-deficit/hyperactivity disorder is characterized by a delay in cortical maturation. Proc. Natl Acad. Sci. USA.

[33] Shaw P, Malek M, Watson B, Sharp W, Evans A (2012). Development of cortical surface area and gyrification in attention-deficit/hyperactivity disorder. Biol. Psychiatry.

[34] van Ewijk H, Heslenfeld DJ, Zwiers MP, Buitelaar JK, Oosterlaan J (2012). Diffusion tensor imaging in attention deficit/hyperactivity disorder: a systematic review and meta-analysis. Neurosci. Biobehav Rev..

[35] Onnink AM (2015). Deviant white matter structure in adults with attention-deficit/hyperactivity disorder points to aberrant myelination and affects neuropsychological performance. Prog. Neuropsychopharmacol. Biol. Psychiatry.

[36] Mostert JC (2016). Characterising resting-state functional connectivity in a large sample of adults with ADHD. Prog. Neuro-Psychopharmacol. Biol. Psychiatry.

[37] Hoogman M (2017). Subcortical brain volume differences in participants with attention deficit hyperactivity disorder in children and adults: a cross-sectional mega-analysis. Lancet Psychiatry.

[38] Castellanos FX (2002). Anatomic magnetic resonance imaging studies of attention-deficit/hyperactivity disorder. Dialogues Clin. Neurosci..

[39] Makris N (2007). Cortical thinning of the attention and executive function networks in adults with attention-deficit/hyperactivity disorder. Cereb. cortex.

[40] Valera EM, Faraone SV, Murray KE, Seidman LJ (2007). Meta-analysis of structural imaging findings in attention-deficit/hyperactivity disorder. Biol. Psychiatry.

[41] Ellison-Wright I, Ellison-Wright Z, Bullmore E (2008). Structural brain change in attention deficit hyperactivity disorder identified by meta-analysis. BMC Psychiatry.

[42] Frodl T, Skokauskas N (2012). Meta-analysis of structural MRI studies in children and adults with attention deficit hyperactivity disorder indicates treatment effects. Acta Psychiatr. Scandinavica.

[43] Nakao T, Radua J, Rubia K, Mataix-Cols D (2011). Gray matter volume abnormalities in ADHD: voxel-based meta-analysis exploring the effects of age and stimulant medication. Am. J. Psychiatry.

[44] Norman LJ (2016). Structural and functional brain abnormalities in attention-deficit/hyperactivity disorder and obsessive-compulsive disorder: a comparative meta-analysis. JAMA Psychiatry.

[45] Bralten J (2016). Voxel-based morphometry analysis reveals frontal brain differences in participants with ADHD and their unaffected siblings. J. Psychiatry Neurosci..

[46] Durston S (2004). Magnetic resonance imaging of boys with attention-deficit/hyperactivity disorder and their unaffected siblings. J. Am. Acad. Child Adolesc. Psychiatry.

[47] Vilgis V, Sun L, Chen J, Silk TJ, Vance A (2016). Global and local grey matter reductions in boys with ADHD combined type and ADHD inattentive type. Psychiatry Res. Neuroimaging.

[48] Shaw P (2006). Longitudinal mapping of cortical thickness and clinical outcome in children and adolescents with attention-deficit/hyperactivity disorder. Arch. Gen. Psychiatry.

[49] Sowell ER (2003). Cortical abnormalities in children and adolescents with attention-deficit hyperactivity disorder. Lancet.

[50] Narr KL (2009). Widespread cortical thinning is a robust anatomical marker for attention-deficit/hyperactivity disorder. J. Am. Acad. Child Adolesc. Psychiatry.

[51] Batty MJ (2010). Cortical gray matter in attention-deficit/hyperactivity disorder: a structural magnetic resonance imaging study. J. Am. Acad. Child Adolesc. Psychiatry.

[52] Schweren LJ (2015). Thinner medial temporal cortex in adolescents with attention-deficit/hyperactivity disorder and the effects of stimulants. J. Am. Acad. Child Adolesc. Psychiatry.

[53] Wolosin SM, Richardson ME, Hennessey JG, Denckla MB, Mostofsky SH (2009). Abnormal cerebral cortex structure in children with ADHD. Hum. Brain Mapp..

[54] de Zeeuw P, Mandl RC, Hulshoff Pol HE, van Engeland H, Durston S (2012). Decreased frontostriatal microstructural organization in attention deficit/hyperactivity disorder. Hum. Brain Mapp..

[55] de Zeeuw P, Zwart F, Schrama R, van Engeland H, Durston S (2012). Prenatal exposure to cigarette smoke or alcohol and cerebellum volume in attention-deficit/hyperactivity disorder and typical development. Transl. Psychiatry.

[56] Ambrosino S, de Zeeuw P, Wierenga LM, van Dijk S, Durston S (2017). What can cortical development in attention-deficit/hyperactivity disorder teach us about the early developmental mechanisms involved?. Cereb. cortex.

[57] Wu ZM (2017). White matter microstructural alterations in children with ADHD: categorical and dimensional perspectives. Neuropsychopharmacology.

[58] Chen L (2016). A systematic review and meta-analysis of tract-based spatial statistics studies regarding attention-deficit/hyperactivity disorder. Neurosci. Biobehav Rev..

[59] Aoki Y, Cortese S, Castellanos FX (2018). Research review: diffusion tensor imaging studies of attention-deficit/hyperactivity disorder: meta-analyses and reflections on head motion. J. Child Psychol. Psychiatry.

[60] Le Bihan D (2003). Looking into the functional architecture of the brain with diffusion MRI. Nat. Rev. Neurosci..

[61] Le Bihan D (2001). Diffusion tensor imaging: concepts and applications. J. Magn. Reson. Imaging..

[62] Yoncheva YN (2016). Mode of anisotropy reveals global diffusion alterations in attention-deficit/hyperactivity disorder. J. Am. Acad. Child Adolesc. Psychiatry.

[63] Cha J (2015). Neural correlates of aggression in medication-naive children with ADHD: multivariate analysis of morphometry and tractography. Neuropsychopharmacology.

[64] Logothetis NK, Pauls J, Augath M, Trinath T, Oeltermann A (2001). Neurophysiological investigation of the basis of the fMRI signal. Nature.

[65] Belliveau JW (1991). Functional mapping of the human visual cortex by magnetic resonance imaging. Science.

[66] Lee, J. H. et al. Global and local fMRI signals driven by neurons defined optogenetically by type and wiring. *Nature* **465**, 788–792 (2010). 10.1038/nature09108 PMC3177305 20473285

[67] Rosen BR, Buckner RL, Dale AM (1998). Event-related functional MRI: past, present, and future. Proc. Natl Acad. Sci. USA.

[68] Huettel, S. A. S. "AW; McCarthy, G." *Functional Magnetic Resonance Imaging*. Sinauer Associates, Inc. (2009).

[69] Cortese, S. et al. Toward systems neuroscience of ADHD: a meta-analysis of 55 fMRI studies. *Am. J. Psychiatry* **169**, 1038–1055 (2012). 10.1176/appi.ajp.2012.11101521 PMC3879048 22983386

[70] Hart H, Radua J, Nakao T, Mataix-Cols D, Rubia K (2013). Meta-analysis of functional magnetic resonance imaging studies of inhibition and attention in attention-deficit/hyperactivity disorder: exploring task-specific, stimulant medication, and age effects. JAMA Psychiatry.

[71] Plichta MM, Scheres A (2014). Ventral-striatal responsiveness during reward anticipation in ADHD and its relation to trait impulsivity in the healthy population: a meta-analytic review of the fMRI literature. Neurosci. Biobehav. Rev..

[72] von Rhein D (2015). Increased neural responses to reward in adolescents and young adults with attention-deficit/hyperactivity disorder and their unaffected siblings. J. Am. Acad. Child Adolesc. Psychiatry.

[73] Cao X (2009). Abnormal resting-state functional connectivity patterns of the putamen in medication-naive children with attention deficit hyperactivity disorder. Brain Res..

[74] Tian L (2006). Altered resting-state functional connectivity patterns of anterior cingulate cortex in adolescents with attention deficit hyperactivity disorder. Neurosci. Lett..

[75] Mennes M (2011). Resting state functional connectivity correlates of inhibitory control in children with attention-deficit/hyperactivity disorder. Front. Psychiatry.

[76] Mills KL (2012). Altered cortico-striatal-thalamic connectivity in relation to spatial working memory capacity in children with ADHD. Front. Psychiatry.

[77] Fox MD, Raichle ME (2007). Spontaneous fluctuations in brain activity observed with functional magnetic resonance imaging. Nat. Rev. Neurosci..

[78] Rubia K, Cubillo A, Woolley J, Brammer MJ, Smith A (2011). Disorder-specific dysfunctions in patients with attention-deficit/hyperactivity disorder compared to patients with obsessive-compulsive disorder during interference inhibition and attention allocation. Hum. Brain Mapp..

[79] Castellanos FX, Proal E (2012). Large-scale brain systems in ADHD: beyond the prefrontal-striatal model. Trends Cogn. Sci..

[80] Posner J, Park C, Wang Z (2014). Connecting the dots: a review of resting connectivity MRI studies in attention-deficit/hyperactivity disorder. Neuropsychol. Rev..

[81] Chabernaud C (2012). Dimensional brain-behavior relationships in children with attention-deficit/hyperactivity disorder. Biol. Psychiatry.

[82] Hulvershorn LA (2014). Abnormal amygdala functional connectivity associated with emotional lability in children with attention-deficit/hyperactivity disorder. J. Am. Acad. Child Adolesc. Psychiatry.

[83] Tan YW (2020). Alterations of cerebral perfusion and functional brain connectivity in medication-naive male adults with attention-deficit/hyperactivity disorder. CNS Neurosci. Ther..

[84] Venkat P, Chopp M, Chen J (2016). New insights into coupling and uncoupling of cerebral blood flow and metabolism in the brain. Croat. Med. J..

[85] Langleben DD (2001). Interhemispheric asymmetry of regional cerebral blood flow in prepubescent boys with attention deficit hyperactivity disorder. Nucl. Med. Commun..

[86] Rubia K, Smith AB, Brammer MJ, Toone B, Taylor E (2005). Abnormal brain activation during inhibition and error detection in medication-naive adolescents with ADHD. Am. J. Psychiatry.

[87] Shaw P, Stringaris A, Nigg J, Leibenluft E (2014). Emotion dysregulation in attention deficit hyperactivity disorder. Am. J. Psychiatry.

[88] MacMaster FP, Carrey N, Sparkes S, Kusumakar V (2003). Proton spectroscopy in medication-free pediatric attention-deficit/hyperactivity disorder. Biol. Psychiatry.

[89] Hesslinger B, Thiel T, Tebartz van Elst L, Hennig J, Ebert D (2001). Attention-deficit disorder in adults with or without hyperactivity: where is the difference? A study in humans using short echo (1)H-magnetic resonance spectroscopy. Neurosci. Lett..

[90] Moore CM (2006). Differences in brain chemistry in children and adolescents with attention deficit hyperactivity disorder with and without comorbid bipolar disorder: a proton magnetic resonance spectroscopy study. Am. J. Psychiatry.

[91] Perlov, E. et al. Reduced cingulate glutamate/glutamine-to-creatine ratios in adult patients with attention deficit/hyperactivity disorder—a magnet resonance spectroscopy study. *J. Psychiatric Res.* **41**, 934–941 (2007). 10.1016/j.jpsychires.2006.12.007 17303167

[92] Perlov E (2009). Spectroscopic findings in attention-deficit/hyperactivity disorder: review and meta-analysis. World J. Biol. Psychiatry.

[93] Cai K (2012). Magnetic resonance imaging of glutamate. Nat. Med..

[94] Crescenzi R (2014). In vivo measurement of glutamate loss is associated with synapse loss in a mouse model of tauopathy. Neuroimage.

[95] Cai K (2013). Mapping glutamate in subcortical brain structures using high-resolution GluCEST MRI. NMR Biomed..

[96] Bagga P (2018). In vivo GluCEST MRI: reproducibility, background contribution and source of glutamate changes in the MPTP model of Parkinson’s disease. Sci. Rep..

[97] Haris M (2013). Imaging of glutamate neurotransmitter alterations in Alzheimer’s disease. NMR Biomed..

[98] Davis KA (2015). Glutamate imaging (GluCEST) lateralizes epileptic foci in nonlesional temporal lobe epilepsy. Sci. Transl. Med..

[99] Roalf DR (2017). Glutamate imaging (GluCEST) reveals lower brain GluCEST contrast in patients on the psychosis spectrum. Mol. Psychiatry.

[100] Chen W (2008). DSM-IV combined type ADHD shows familial association with sibling trait scores: a sampling strategy for QTL linkage. Am. J. Med. Genet. B Neuropsychiatr. Genet..

[101] Gizer IR, Ficks C, Waldman ID (2009). Candidate gene studies of ADHD: a meta-analytic review. Hum. Genet..

[102] Faraone SV (2005). Molecular genetics of attention-deficit/hyperactivity disorder. Biol. Psychiatry.

[103] Li D, Sham PC, Owen MJ, He L (2006). Meta-analysis shows significant association between dopamine system genes and attention deficit hyperactivity disorder (ADHD). Hum. Mol. Genet..

[104] Larsson H (2013). Genetic and environmental influences on adult attention deficit hyperactivity disorder symptoms: a large Swedish population-based study of twins. Psychol. Med..

[105] Franke B, Neale BM, Faraone SV (2009). Genome-wide association studies in ADHD. Hum. Genet..

[106] Brookes KJ (2006). A common haplotype of the dopamine transporter gene associated with attention-deficit/hyperactivity disorder and interacting with maternal use of alcohol during pregnancy. Arch. Gen. Psychiatry.

[107] Zhou K (2008). Meta-analysis of genome-wide linkage scans of attention deficit hyperactivity disorder. Am. J. Med. Genet. Part B, Neuropsychiatr. Genet..

[108] Rivero O (2015). Cadherin-13, a risk gene for ADHD and comorbid disorders, impacts GABAergic function in hippocampus and cognition. Transl. Psychiatry.

[109] Ribases M (2011). Contribution of LPHN3 to the genetic susceptibility to ADHD in adulthood: a replication study. Genes Brain Behav..

[110] Williams NM (2010). Rare chromosomal deletions and duplications in attention-deficit hyperactivity disorder: a genome-wide analysis. Lancet.

[111] Lesch KP (2011). Genome-wide copy number variation analysis in attention-deficit/hyperactivity disorder: association with neuropeptide Y gene dosage in an extended pedigree. Mol. Psychiatry.

[112] Hawi Z (2015). The molecular genetic architecture of attention deficit hyperactivity disorder. Mol. Psychiatry.

[113] Yang L (2013). Polygenic transmission and complex neuro developmental network for attention deficit hyperactivity disorder: genome-wide association study of both common and rare variants. Am. J. Med. Genet. B Neuropsychiatr. Genet..

[114] Li Z, Chang SH, Zhang LY, Gao L, Wang J (2014). Molecular genetic studies of ADHD and its candidate genes: a review. Psychiatry Res..

[115] Schreiweis C (2014). Humanized Foxp2 accelerates learning by enhancing transitions from declarative to procedural performance. Proc. Natl Acad. Sci. USA.

[116] Tsui, D., Vessey, J. P., Tomita, H., Kaplan, D. R. & Miller, F. D. FoxP2 regulates neurogenesis during embryonic cortical development. *J. Neurosci*. **33**, 244–258 (2013). 10.1523/JNEUROSCI.1665-12.2013 PMC6618635 23283338

[117] Breiderhoff T (2013). Sortilin-related receptor SORCS3 is a postsynaptic modulator of synaptic depression and fear extinction. PLoS ONE.

[118] Mortensen OV, Larsen MB, Prasad BM, Amara SG (2008). Genetic complementation screen identifies a mitogen-activated protein kinase phosphatase, MKP3, as a regulator of dopamine transporter trafficking. Mol. Biol. Cell.

[119] Li C, Scott DA, Hatch E, Tian X, Mansour SL (2007). Dusp6 (Mkp3) is a negative feedback regulator of FGF-stimulated ERK signaling during mouse development. Development.

[120] Carithers LJ (2015). A novel approach to high-quality postmortem tissue procurement: the GTEx project. Biopreserv. Biobank.

[121] Peper JS, Brouwer RM, Boomsma DI, Kahn RS, Hulshoff, Pol HE (2007). Genetic influences on human brain structure: a review of brain imaging studies in twins. Hum. Brain Mapp..

[122] McKay DR (2014). Influence of age, sex and genetic factors on the human brain. Brain Imaging Behav..

[123] Kochunov P (2015). Heritability of fractional anisotropy in human white matter: a comparison of Human Connectome Project and ENIGMA-DTI data. NeuroImage.

[124] Glahn DC (2010). Genetic control over the resting brain. Proc. Natl Acad. Sci. USA.

[125] Blokland GA (2014). Genetic effects on the cerebellar role in working memory: same brain, different genes?. NeuroImage.

[126] Gallo EF, Posner J (2016). Moving towards causality in attention-deficit hyperactivity disorder: overview of neural and genetic mechanisms. Lancet Psychiatry.

[127] Shang CY, Lin HY, Tseng WY, Gau SS (2018). A haplotype of the dopamine transporter gene modulates regional homogeneity, gray matter volume, and visual memory in children with attention-deficit/hyperactivity disorder. Psychol. Med..

[128] Pribilova N (2016). Long term pharmacotherapy by methylfenidate or atomoxetine DAT 1 10/10 ADHD children in correlation with results of the imaging methods. Neuro Endocrinol. Lett..

[129] Fernandez-Jaen A (2015). Cortical thickness differences in the prefrontal cortex in children and adolescents with ADHD in relation to dopamine transporter (DAT1) genotype. Psychiatry Res..

[130] Onnink AM (2016). Enlarged striatal volume in adults with ADHD carrying the 9-6 haplotype of the dopamine transporter gene DAT1. J. Neural Transm..

[131] Fernandez-Jaen A (2018). Cingulate cortical thickness and dopamine transporter (DAT1) genotype in children and adolescents With ADHD. J. Atten. Disord..

[132] Shaw P (2007). Polymorphisms of the dopamine D4 receptor, clinical outcome, and cortical structure in attention-deficit/hyperactivity disorder. Arch. Gen. Psychiatry.

[133] Monuteaux MC (2008). A preliminary study of dopamine D4 receptor genotype and structural brain alterations in adults with ADHD. Am. J. Med. Genet. B Neuropsychiatr. Genet..

[134] Schweren LJ (2016). Age and DRD4 genotype moderate associations between stimulant treatment history and cortex structure in attention-deficit/hyperactivity disorder. J. Am. Acad. Child Adolesc. Psychiatry.

[135] Villemonteix T (2015). Structural correlates of COMT Val158Met polymorphism in childhood ADHD: a voxel-based morphometry study. World J. Biol. Psychiatry.

[136] Shimada K (2017). Ethnic differences in COMT genetic effects on striatal grey matter alterations associated with childhood ADHD: a voxel-based morphometry study in a Japanese sample. World J. Biol. Psychiatry.

[137] Park S (2015). Associations between serotonin transporter gene (SLC6A4) methylation and clinical characteristics and cortical thickness in children with ADHD. Psychol. Med..

[138] Soderqvist S (2010). The SNAP25 gene is linked to working memory capacity and maturation of the posterior cingulate cortex during childhood. Biol. Psychiatry.

[139] Li J (2015). The cortical surface area of the insula mediates the effect of DBH rs7040170 on novelty seeking. NeuroImage.

[140] Hong SB (2015). COMT genotype affects brain white matter pathways in attention-deficit/hyperactivity disorder. Hum. Brain Mapp..

[141] Kabukcu Basay B (2016). White matter alterations related to attention-deficit hyperactivity disorder and COMT val(158)met polymorphism: children with valine homozygote attention-deficit hyperactivity disorder have altered white matter connectivity in the right cingulum (cingulate gyrus). Neuropsychiatr. Dis. Treat..

[142] Takeuchi H (2015). Cognitive and neural correlates of the 5-repeat allele of the dopamine D4 receptor gene in a population lacking the 7-repeat allele. NeuroImage.

[143] Paloyelis Y, Mehta MA, Faraone SV, Asherson P, Kuntsi J (2012). Striatal sensitivity during reward processing in attention-deficit/hyperactivity disorder. J. Am. Acad. Child Adolesc. Psychiatry.

[144] Bedard AC (2010). Dopamine transporter gene variation modulates activation of striatum in youth with ADHD. NeuroImage.

[145] van Rooij D (2015). Altered neural connectivity during response inhibition in adolescents with attention-deficit/hyperactivity disorder and their unaffected siblings. NeuroImage Clin..

[146] Sokolova E (2015). Causal discovery in an adult ADHD data set suggests indirect link between DAT1 genetic variants and striatal brain activation during reward processing. Am. J. Med. Genet. B Neuropsychiatr. Genet..

[147] Ko, C. H. et al. The altered brain activation of phonological working memory, dual tasking, and distraction among participants with adult ADHD and the effect of the MAOA polymorphism. *J. Atten. Disord.* **22**, 240–9 (2018).. 10.1177/1087054715572609 25777072

[148] Hoogman M (2013). The dopamine transporter haplotype and reward-related striatal responses in adult ADHD. Eur. Neuropsychopharmacol..

[149] Qian A (2018). Effects of the 2-repeat allele of the DRD4 gene on neural networks associated with the prefrontal cortex in children with ADHD. Front. Hum. Neurosci..

[150] Qian A (2018). Dopamine D4 receptor gene associated with the frontal-striatal-cerebellar loop in children with ADHD: a resting-state fMRI study. Neurosci. Bull..

[151] Kim JI, Yoo JH, Kim D, Jeong B, Kim BN (2018). The effects of GRIN2B and DRD4 gene variants on local functional connectivity in attention-deficit/hyperactivity disorder. Brain Imaging Behav..

[152] van der Meer D (2017). Effects of dopaminergic genes, prenatal adversities, and their interaction on attention-deficit/hyperactivity disorder and neural correlates of response inhibition. J. Psychiatry Neurosci..

[153] Richards JS (2016). Adolescent behavioral and neural reward sensitivity: a test of the differential susceptibility theory. Transl. Psychiatry.

[154] Ercan ES (2016). Decreasing ADHD phenotypic heterogeneity: searching for neurobiological underpinnings of the restrictive inattentive phenotype. Eur. Child Adolesc. Psychiatry.

[155] Gilsbach S (2012). Effects of the DRD4 genotype on neural networks associated with executive functions in children and adolescents. Dev. Cogn. Neurosci..

[156] Pineau G (2019). Dopamine transporter genotype modulates brain activity during a working memory task in children with ADHD. Res. Dev. Disabil..

[157] Stollstorff M (2010). Neural response to working memory load varies by dopamine transporter genotype in children. NeuroImage.

[158] van Rooij D (2015). Influence of DAT1 and COMT variants on neural activation during response inhibition in adolescents with attention-deficit/hyperactivity disorder and healthy controls. Psychol. Med..

[159] van Rooij D (2015). Variation in serotonin neurotransmission genes affects neural activation during response inhibition in adolescents and young adults with ADHD and healthy controls. World J. Biol. Psychiatry.

[160] Mizuno Y (2017). Catechol-O-methyltransferase polymorphism is associated with the cortico-cerebellar functional connectivity of executive function in children with attention-deficit/hyperactivity disorder. Sci. Rep..

[161] Brown AB (2010). Effect of dopamine transporter gene (SLC6A3) variation on dorsal anterior cingulate function in attention-deficit/hyperactivity disorder. Am. J. Med. Genet. B Neuropsychiatr. Genet..

[162] Wang, C. et al. The impact of SNAP25 on brain functional connectivity density and working memory in ADHD. *Biol. Psychol*. **138**, 35–40 (2018). 10.1016/j.biopsycho.2018.08.005 30092259

[163] Nymberg C (2013). Neural mechanisms of attention-deficit/hyperactivity disorder symptoms are stratified by MAOA genotype. Biol. Psychiatry.

[164] Inci Kenar AN (2016). Relationship between the DAT1 gene and the effects of methylphenidate administration in adult attention deficit hyperactivity disorder: a magnetic resonance spectroscopy study. Eur. Rev. Med. Pharmacol. Sci..

[165] Unal GA, Inci Kenar AN, Tepeli E, Kiroglu Y, Herken H (2016). Relationship between the SNAP-25 gene and the effects of methylphenidate on the anterior cingulate cortex of patients with adult attention deficit hyperactivity disorder: a magnetic resonance spectroscopy study. Eur. Rev. Med. Pharmacol. Sci..

[166] Basay O (2016). The impact of synapsin III gene on the neurometabolite level alterations after single-dose methylphenidate in attention-deficit hyperactivity disorder patients. Neuropsychiatr. Dis. Treat..

[167] Ozturk O (2016). The effect of single dose methylphenidate on neurometabolites according to COMT gene Val158Met polymorphism in the patient with attention deficit hyperactivity disorder: a study using magnetic resonance spectroscopy. Clin. Psychopharmacol. Neurosci..

[168] Davidovitch M, Koren G, Fund N, Shrem M, Porath A (2017). Challenges in defining the rates of ADHD diagnosis and treatment: trends over the last decade. BMC Pediatrics.

[169] Bruchmuller K, Margraf J, Schneider S (2012). Is ADHD diagnosed in accord with diagnostic criteria? Overdiagnosis and influence of client gender on diagnosis. J. Consult Clin. Psychol..

[170] Fresson M, Meulemans T, Dardenne B, Geurten M (2019). Overdiagnosis of ADHD in boys: stereotype impact on neuropsychological assessment. Appl. Neuropsychol. Child.

[171] Thomas R, Sanders S, Doust J, Beller E, Glasziou P (2015). Prevalence of attention-deficit/hyperactivity disorder: a systematic review and meta-analysis. Pediatrics.

[172] Hamed AM, Kauer AJ, Stevens HE (2015). Why the diagnosis of attention deficit hyperactivity disorder matters. Front. Psychiatry.

[173] Faraone SV, Larsson H (2019). Genetics of attention deficit hyperactivity disorder. Mol. Psychiatry.

[174] Osland, S. T., Steeves, T. D. L. & Pringsheim, T. Pharmacological treatment for attention deficit hyperactivity disorder (ADHD) in children with comorbid tic disorders. *Cochrane Database Syst Rev.* **6**, CD007990 (2018). 10.1002/14651858.CD007990.pub3 PMC6513283 29944175

[175] Durston S (2005). Differential effects of DRD4 and DAT1 genotype on fronto-striatal gray matter volumes in a sample of subjects with attention deficit hyperactivity disorder, their unaffected siblings, and controls. Mol Psychiatry.

[176] Shook, D. & et al. Effect of dopamine transporter genotype on caudate volume in childhood ADHD and controls. American journal of medical genetics Part B, Neuropsychiatric genetics : the official publication of the International Society of Psychiatric Genetics. 156B, 28–35 (2011).. 10.1002/ajmg.b.31132 PMC3010298 20957668

[177] Castellanos FX (1998). Lack of an association between a dopamine-4 receptor polymorphism and attentiondeficit/hyperactivity disorder: genetic and brain morphometric analyses. Mol Psychiatry..

[178] van der Meer D (2015). Brain Correlates of the Interaction Between 5-HTTLPR and Psychosocial Stress Mediating Attention Deficit Hyperactivity Disorder Severity. Am J Psychiatry..

[179] van der Meer D (2016). Interplay between stress response genes associated with attention deficithyperactivity disorder and brain volume. Genes Brain Behav..

[180] Bobb, A, J. et al. Support for association between ADHD and two candidate genes: NET1 andDRD1. American journal of medical genetics Part B, Neuropsychiatric genetics : the official publication of the International Society of Psychiatric Genetics. 2005; 134B: 67–72. 10.1002/ajmg.b.30142 15717291

[181] van Ewijk H (2017). Female-specific association of NOS1 genotype with white matter microstructure in ADHD patients and controls. J Child Psychol Psychiatry.

[182] Park S (2013). White-matter connectivity and methylphenidate-induced changes in attentional performance according to alpha2A-adrenergic receptor gene polymorphisms in Korean children with attention-deficit hyperactivity disorder.. J Neuropsychiatry Clin Neurosci..

[183] Brown AB (2011). Relationship of DAT1 and adult ADHD to task-positive and task-negative working memory networks.. Psychiatry Res..

[184] Braet W (2011). fMRI activation during response inhibition and error processing: the role of the DAT1 gene in typically developing adolescents and those diagnosed with ADHD. Neuropsychologia.

[185] Durston S (2008). Dopamine transporter genotype conveys familial risk of attention deficit/hyperactivity disorder through striatal activation. J Am Acad Child Adolesc Psychiatry.

[186] Hoogman M (2011). Nitric oxide synthase genotype modulation of impulsivity and ventral striatal activity in adult ADHD patients and healthy comparison subjects. Am J Psychiatry.

[187] Fontana BD (2019). Zebrafish models for attention deficit hyperactivity disorder (ADHD). Neurosci. Biobehav. Rev..

[188] Tripp G, Wickens JR (2009). Neurobiology of ADHD. Neuropharmacology.

